# Rare variants in drug target genes contributing to complex diseases, phenome-wide

**DOI:** 10.1038/s41598-018-22834-4

**Published:** 2018-03-15

**Authors:** Shefali Setia Verma, Navya Josyula, Anurag Verma, Xinyuan Zhang, Yogasudha Veturi, Frederick E. Dewey, Dustin N. Hartzel, Daniel R. Lavage, Joe Leader, Marylyn D. Ritchie, Sarah A. Pendergrass

**Affiliations:** 1 Perelman School of Medicine, Department of Genetics, University of Pennsylvania, Philadelphia, PA 19104 USA; 2 Biomedical and Translational Informatics Institute, Geisinger, Danville, PA 17221 USA; 3 Phenomic Analytics and Clinical Data Core, Geisinger, Danville, PA USA; 4 Regeneron Genetics Center, Tarrytown, NY 10591 USA

## Abstract

The DrugBank database consists of ~800 genes that are well characterized drug targets. This list of genes is a useful resource for association testing. For example, loss of function (LOF) genetic variation has the potential to mimic the effect of drugs, and high impact variation in these genes can impact downstream traits. Identifying novel associations between genetic variation in these genes and a range of diseases can also uncover new uses for the drugs that target these genes. Phenome Wide Association Studies (PheWAS) have been successful in identifying genetic associations across hundreds of thousands of diseases. We have conducted a novel gene based PheWAS to test the effect of rare variants in DrugBank genes, evaluating associations between these genes and more than 500 quantitative and dichotomous phenotypes. We used whole exome sequencing data from 38,568 samples in Geisinger MyCode Community Health Initiative. We evaluated the results of this study when binning rare variants using various filters based on potential functional impact. We identified multiple novel associations, and the majority of the significant associations were driven by functionally annotated variation. Overall, this study provides a sweeping exploration of rare variant associations within functionally relevant genes across a wide range of diagnoses.

## Introduction

While genome wide association studies (GWAS) and Phenome Wide Association Studies (PheWAS) studies have identified novel and replicating associations for many common genetic variants and complex traits^1–5^, rare variation coupled with comprehensive PheWAS associations are only beginning to be explored. Rare variation studies have the potential for uncovering novel and informative relationships between genetic architecture and common diseases, increasing our understanding of biological mechanisms as well as identifying key targets for drug development^6^. For example, gain of function rare variation in the lipid pathway gene *PCSK9* is associated with familial hypercholesterolemia, while loss of function mutations lead to lower levels of LDL-cholesterol^7^. Thus, drugs have now been developed that target *PCSK9* to lower LDL-cholesterol levels^8,9^. In addition, rare genetic variation can also perturb biological networks, impacting the risk and protection for conditions as well as impacting quantitative traits such as clinical laboratory measures. Further, risk or protective impact on one trait may be reversed for another trait, due to antagonistic pleiotropy. Finally, contrasting protective and risk associations for specific genes can highlight potential drug side effects^10^. With PheWAS, we can interrogate a wide array of quantitative clinical laboratory measures and dichotomous diagnoses across rare variation, including potentially functionally high impact rare variation, across many genes^11–13^ to identify new hypotheses for gene function.

Using rare-variant collapsing approaches and choosing rare variants based on functional category has been shown to be of importance^14,15^. For example, loss of function (LOF) variants result in the truncation or lack of translation of a protein, and thus have the potential for a very strong impact on downstream phenotypes. Functional annotation of variations can be obtained from several predictive and analytical tools^16–18^. Binning these filtered variants and testing them against multiple phenotypes has the potential for different insights depending on how the variants are filtered.

The DrugBank database (version 4.0)^19^ is a resource with extremely well characterized genes and the drugs that target those genes. In this study, we performed a PheWAS using ~800 unique genes from the DrugBank database evaluating comprehensive associations between these genes and 541 diagnoses and 35 quantitative clinical lab measures using a gene burden-based approach. For this study, we used whole exome sequencing data from 38,568 unrelated European American adults (>18 years of age) from the MyCode Community Health Initiative, from Geisinger a large health care provider^20^. To explore how results changed depending on different methods for filtering rare variants, we used several approaches: all rare variants within the DrugBank specified genes, as well as LOF and non-synonymous variants via different predicting algorithms and filters. We also contrasted our results with burden based association testing of all rare variants that lacked functional annotation. Our goal was to identify (1) the impact of LOF variants on disease risk, (2) protective effect of variants in these genes, (3) cross-phenotype associations for these targeted genes.

We identified novel associations between these genes and diagnoses and quantitative clinical lab measures, identifying many associations that are supported by the known biological impact of these genes. We contrasted our results with the known function of these genes in the context of drugs and the diagnoses these genes target, as well as evaluated cross phenotype associations. We also evaluated associations where variants were filtered by functional impact. Overall, we have identified novel genetic associations providing new insights across many phenotypes for a series of high impact genes, with the additional context of gene function, genetic pathways, the functional impact of genetic variation, and potential pleiotropy.

## Results

For the results of associations between various low frequency variant filtering methods, for 797 DrugBank genes using whole exome sequencing data, we found a total of 91 results that passed the Bonferroni threshold (P-value = 1.08e − 07); all 91 results passing this threshold are in Supplementary Table 1. Table 1 also lists the most potentially novel gene-phenotype associations for clinical lab and diagnosis codes of our study.

**Table 1:** Potential novel associations from PheWAS analyses.

Gene	Phenotype	#Samples	Filter Type	Beta	OR	SE	P-value
*GLCCI1*	WBC Counts	36587	Functional Annotation Filter 2	−0.32	0.72	0.04	2.33E − 13
*SLC12A3*	Potassium	36039	Functional Annotation Filter 3	−0.07	0.93	0.01	5.91E − 07
*PTGR2*	Abnormal glucose tolerance of mother	38313	Functional Annotation Filter 2	4.02	0.68	55.7	5.48E − 09
*PTGR2*	Abnormal glucose tolerance of mother	38313	Functional Annotation Filter 3	3.19	0.61	24.28	1.70E − 07
*PTGR2*	Abnormal glucose tolerance of mother	38313	All Variants	2.9	0.56	18.17	3.18E − 07
*PTGR2*	Abnormal glucose tolerance of mother	38313	Functional Annotation Filter 1	3.04	0.6	20.9	5.52E − 07
*FOS*	Sensorineural hearing loss, unspecified	36864	Functional Annotation Filter 2	3.03	20.67	0.56	6.73E − 08
*FOS*	Sensorineural hearing loss, unspecified	36864	All Variants	1.74	5.70	0.38	5.71E − 06
*FOS*	Sensorineural hearing loss, unspecified	36864	Functional Annotation Filter 3	1.84	6.30	0.47	9.45E − 05
*FOS*	Sensorineural hearing loss, unspecified	36864	Functional Annotation Filter 1	1.81	6.11	0.47	1.29E − 04
*ATF7*	Overweight	35809	Functional Annotation Filter 2	2.73	15.33	0.54	3.69E − 07
*ATF7*	Overweight	35809	Functional Annotation Filter 1	1.96	7.09	0.43	4.19E − 06
*ATF7*	Overweight	35809	Functional Annotation Filter 3	1.94	6.95	0.44	9.59E − 06
*ATF7*	Overweight	35809	All Variants	1.52	4.57	0.41	1.85E − 04

The two most significant results of this study are associations between genes and phenotypes where the impact of loss of function highly relates to the known function of these genes. For example, the top result from the diagnosis codes analysis observed in the functional annotation filter category 2 was an association between the calcium-sensing receptor gene (*CASR*) and the diagnosis of “hypercalcemia” (ICD-9 275.42, P-value = 1.34e − 22, beta = 3.89, functional annotation filter 2), Supplemental Table 1. *CASR* plays an essential role in calcium homeostasis and is expressed mostly in kidneys and parathyroid glands. Mutations in *CASR* lead to familial hypocalciuric hypercalcemia (FHH)^21^. In our study, this association was Bonferroni significant using all four functionally annotated filter categories with the most significant result for functional annotation filter 2. Associations between *CASR* and hypercalcemia were least significant via the ‘all variants’ filtering category (functional annotation filter 1). This suggests the effect of association is impacted more strongly by functionally annotated variants in the *CASR* gene rather than non-functionally annotated variants. The diagnoses used in drug treatments that target *CASR* are hyperparathyroidism, bone destruction, chronic kidney disease with secondary hyperparathyroidism and impaired renal function. The most significant result from the clinical laboratory measurement analyses was the association between the gene *GPT* and alanine aminotransferase levels (P-value = 3.29e − 83; Beta = −0.64; functional annotation filter 1), Supplemental Table 1. The *GPT* gene encodes the enzyme glutamate-pyruvate transaminase 1, also known as cytosolic alanine aminotransferase. Comparative analyses of GPT with alanine aminotransferase among the four filter categories suggests that functionally annotated variants have a larger impact on phenotypic variation than non-functionally annotated variants.

### Associations with Clinical Lab Measures

**Figure 1:**
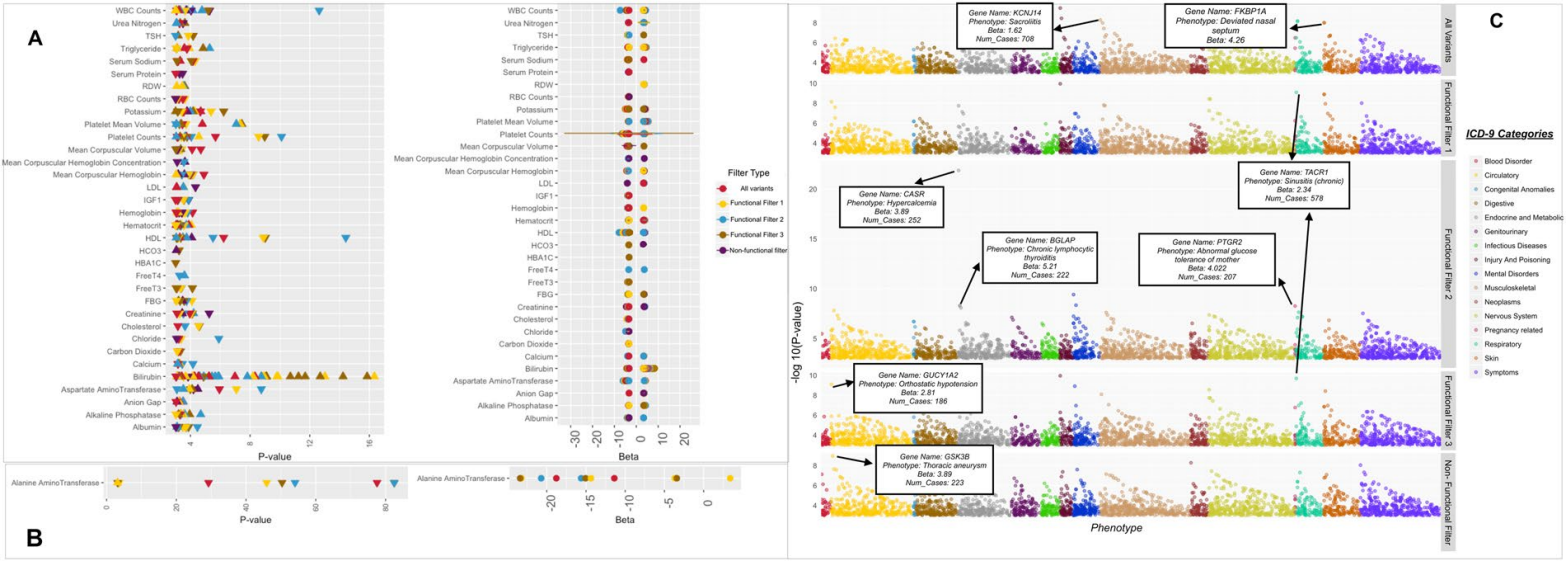
Phewas-view plot of clinical laboratory measures. (**A**) Represents clinical laboratory measurement gene based associations for results with P-value < 0.001. The Y-axis lists all phenotypes. Triangles represent the –log10 P-value of associations on the left, with normalized beta in on the right with standard error bars. Points are colored based on the filter category. The direction of the triangle corresponds to the direction of effect: up is positive, down is negative. The results for alanine aminotransferase (ALT) are plotted separately in (**B**) due to the significance of tshe results on a different scale from the rest of the results. (**C**) Is a Manhattan plot of associations with P-value < 0.001 for ICD-9 code based case/control diagnoses. The x-axis corresponds to ICD-9 category and y-axis corresponds to the −log10(P-value) of the association and the points are colored based on the ICD-9 category. Within each diagnosis category the plotted points are ordered from most significant associations to the least significant associations.

To show the overall landscape of results for the clinical lab measures for both highly significant and more potentially suggestive associations, we plotted all results below an exploratory P-value of 0.001 for clinical labs in Fig. 1A and B. There were 197 unique gene-phenotype combinations.

We found a total of 21 Bonferroni significant associations with quantitative laboratory measurements from a total of 5 unique gene-phenotype combinations (due to significant associations for the same gene-phenotype combinations from multiple ways of filtering rare variants). Of these, 20 associations are supported by the known function of these genes, impact on respective associated phenotypes through previously reported common genetic variation results, and through other existing biological knowledge. We identified 1 potentially novel association without considerable previous biological knowledge relating the gene to the phenotype.

A total of 14 associations out of all Bonferroni significant results were for bilirubin levels and represented alternative splicing forms of *UGT1A* gene. The *UGT1A* gene is known for highly significant associations with bilirubin for common frequency variants^22^,^23–25^. This gene family encodes enzyme UDP-glucuronosyltransferase, which converts toxic bilirubin into non-toxic form^26^. Of note, all the highly significant associations *only* included functionally annotated filtered and all variants categories. No associations in the non-functionally annotated category reached Bonferroni significance.

We also identified associations between *GOT1* and aspartate aminotransferase levels^27^ (in functional annotation Filter 1 and 2). *GOT1* is known as Glutamic-Oxaloacetic Transaminase 1 (also known as aspartate aminotransferase), therefore its association with aspartate aminotransferase levels in serum reflects knowledge of this gene.

We found a highly significant association between *TUBB1* and platelet counts (P-value = 7.85e − 11; normalized beta = −6.50; functional annotation Filter 2) that was also Bonferroni significant via functional annotation filters 1 and 3. *TUBB1* was also associated with the related mean platelet volume (P-value = 3.57e − 08; Normalized Beta = 5.51 in functional annotation Filter 3), but with a positive direction of effect. The *TUBB1* gene is highly expressed in platelets and megakaryocytes and has been inferred to be involved in proplatelet production and platelet release^28,29^. The *TUBB1* protein is one of the two core families to form microtubules. Loss of function in the *TUBB1* gene inhibits platelet release, which results in decrease in platelet counts. This supports our finding of a negative direction of effect in our association with platelet counts, indicating that an enrichment in functionally annotated mutations leads to a decrease in platelet counts. It has also been shown that mutation in this gene is associated with autosomal dominant macrothrombocytopenia, with both a reduction in platelet counts as well as an increase in platelet volume. This is also consistent with the observation in our study that loss of function of this gene is positively associated with platelet mean volume (a measure of the average size of platelets in blood)^30,31^.

Another association from our study is between *GLCCI1* gene (using functional annotation filter 2) with white blood cell (WBC) counts (Table 1). Common frequency SNPs in the *GLCCI1* gene have been previously associated with asthma^32,33^, but not WBC counts. High WBC counts are a reflection of inflammation, and higher WBC counts are observed in patients with severe allergy and asthma^34^. A direct connection between this gene and WBC levels is not known.

### Associations with Clinical Diagnoses

Of the 70 Bonferroni significant associations with ICD-9 code based diagnoses, there were 60 unique gene-phenotype combinations. Of these, 14 associations are closely related to the known function of these genes, and 57 associations are more novel with respect to existing understanding of these genes.

For ICD-9 code associations, we have highlighted some of the key results of these associations in the Manhattan plot of Fig. 1C. Among the top associations is the gene *TACR1* associated with *chronic sinusitis* (ICD-9 473.9; P-value = 2.01e − 10; beta = 2.34; functional annotation filter 3). The *TACR1* gene is from the family of tachykinin receptors that are characterized by the interactions with G-proteins. Other G-protein receptors such as *I*_*KACH*_*, GNB2* are linked to some other forms of sinusitis^35,36^ but this association between functional annotation mutations in *TACR1* and chronic sinusitis is novel. The drug aprepitant is a known target drug for gene *TACR1*, and is an antagonist of the receptor. It is used to treat nausea and vomiting symptoms caused by chemotherapy treatment for cancer^37,38^. This novel association among functionally annotated mutations in *TACR1* and chronic sinusitis warrants further investigation to understand what impact this drug may have in relation to sinusitis, including a potential side effect of tachykinin receptor blocking through the use of this drug.

In our study, we also identified functionally annotated variants in the *PTGR2* gene (from all categories where functionally annotated mutations were tested) with “abnormal glucose intolerance of mother”. These association results are below the Bonferroni cutoff in all 4 categories where functionally annotated variants are included. For these associations, we observed the highest odds ratio of 55.70 in functional annotation filter 2 and lowest OR of 18.17 in the all variants category. These results are shown in Table 1. This association is also not p previously reported. Gene *PTGR2* also showed a Bonferroni significant association in the non-functionally annotated category with the diagnosis of ICD-9 code 309.28 (anxiety and depression).

### Overall Trends of Results Across Variant Filtering Approaches

**Figure 2:**
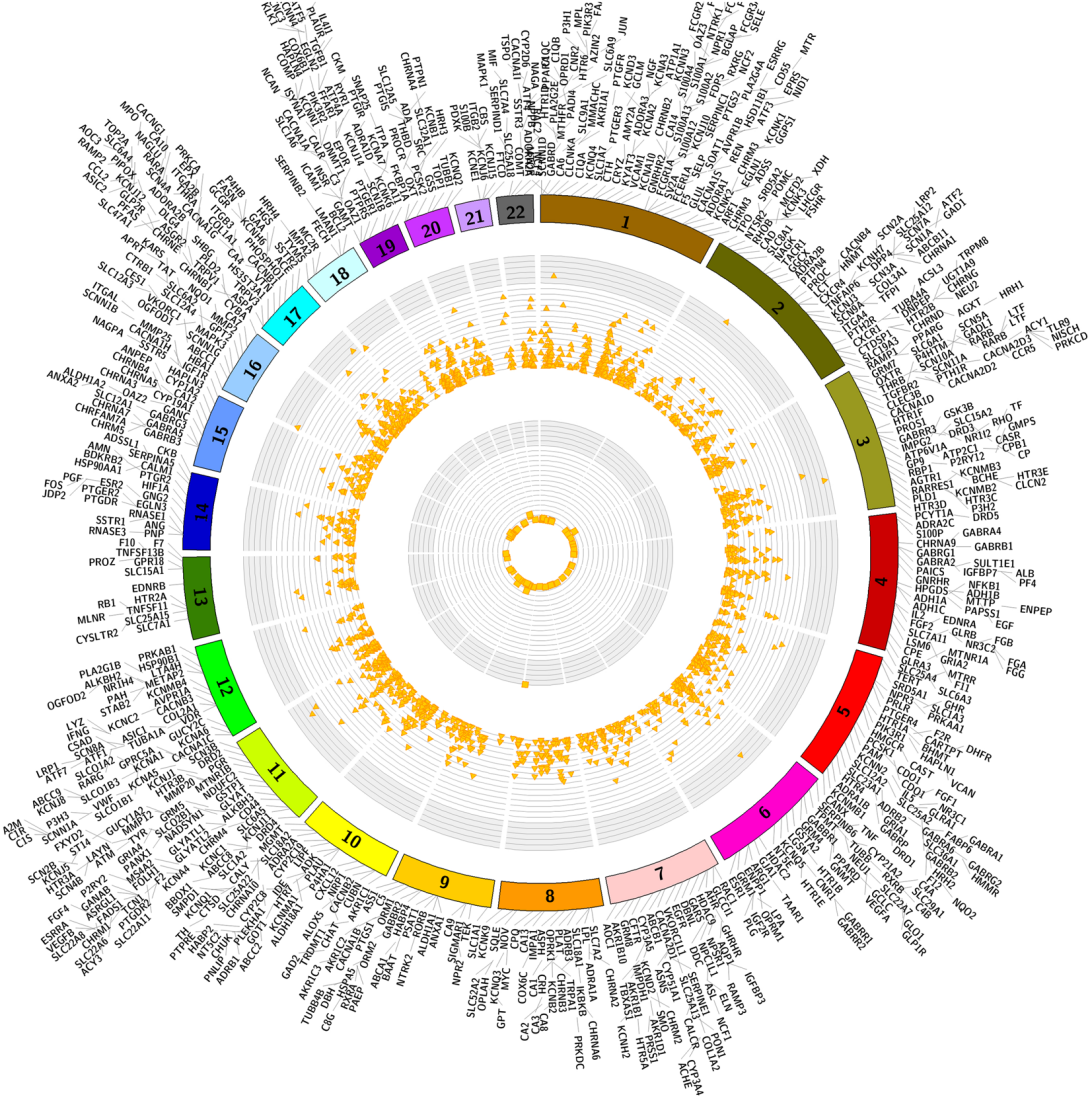
Circos plot of –log 10(P-value) association results by chromosome from ICD-9 codes (outer circle, points represented as triangles) and clinical laboratory values (points represented as squares) for results with P-value < 0.001. The genes are labeled at their respective chromosome base pair location boundaries. Yellow points represent results from functional annotation filter 1 category. The axis on both plots is same and goes from 0 to 22 (−log10 P-value).

A focus of this study was comparing and contrasting the results of gene-based comprehensive associations across a wide range of phenotypes when using a range of approaches for filtering rare variants. Figure 2 below shows a circos plots representing all results with P-values less than 0.001 from the functional annotation filter 2 category for results from associations with ICD-9 based case/control status as well as the quantitative clinical lab measures. Plots for other functionally annotated filters, all variants and non- functionally annotated filter categories are shown in Supplementary Figures 1, 2, 3 and 4. We have presented results from each of the functional annotation filters in separate colors to exemplify the differences and similarities observed in these analyses.

For ICD-9 based diagnoses, the results from functional annotation filter 2 had the least number of associations that were significant at an alpha level of 0.001. However, this filter also had the most significant association of the entire study (*CASR and* hypercalcemia), as well as the most significant associations for ICD-9 based diagnoses. Also for ICD-9 based diagnoses the non-functionally annotated category showed the highest number of results at P-value < 0.001, but the lowest P-value was 1.08e − 09 for the gene *GSK3B* associated with the diagnosis “thoracic aneurysm”, ICD-9 441.2. In the other functionally annotated filter categories for associations with diagnoses, the lowest P-value was 1e − 10, and in the non-functionally annotated category the lowest P-value was 1e − 08.

For clinical lab measures associations, filtering rare variants for functional annotation showed more number of highly statistically significant results than non-filtering by functional annotation (all variants and non-functional annotation filter categories), with the top result for functional annotation filter 1, 2 and 3 was for *GPT* associated with alanine aminotransferase levels (P-value = 3.29e − 83).

**Figure 3:**
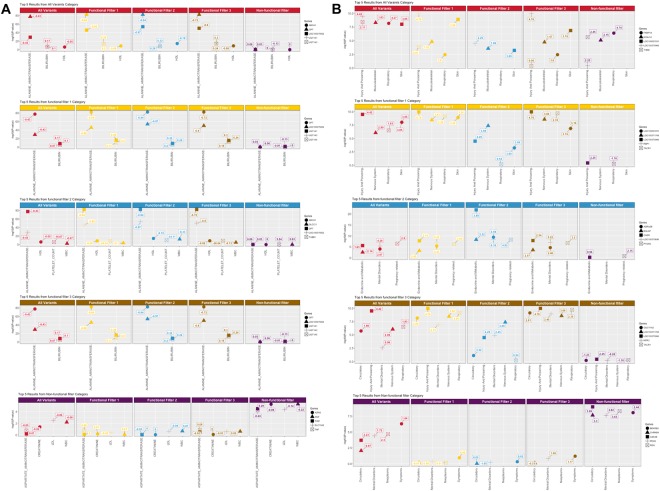
(**A**) Shows the top 5 results from each rare variant filtering strategy and corresponding –log10(P-value) and magnitude and direction of effect (boxes linked to the data points) from other filtering strategies for the same gene-phenotype associations for clinical lab measures. The x axis shows the clinical lab name and y-axis shows the –log10(p-value). Beta coefficient are represented by numbers next to points which are color coded by filter category and shape corresponds to gene name. (**B**) Shows the top 5 results from each rare variant filtering strategy and corresponding –log10(P-value) and magnitude and direction of effect (boxes linked to the data points) from other filtering strategies for the same gene-phenotype associations for ICD-9 based diagnoses. The x axis shows the general ICD-9 category the diagnosis was grouped into and y-axis shows the –log10(p-value). Beta coefficients are represented by numbers next to points, are color coded by filter category, and the shape corresponds to the specific gene listed in the legend.

To compare and contrast the effect of associations that are significant for one rare variant filter and marginally or not significant in other filters, we picked the top 5 genetic associations from each functional annotation filter and plotted the P-values of the same gene and phenotype associations from the other functional annotation categories. These results are shown in Fig. 3A and B. For example, for the ICD-9 diagnosis based associations, the most significant association in the all variants category was between the gene *THBD* and the diagnosis “other closed fractures of distal end of radius” (ICD-9 code 813.42, P-value = 3.54e − 09). This result seems to be influenced mainly by non-functionally annotated variants as it is (a) significant in all variants, (b) not statistically significant for the non-functionally annotated filter (P-value = 2.23e − 06) and (c) not significant in the functional annotation filter categories.

The association between gene *ADRA2B* and “alcohol abuse” ICD-9 305.00 (mental disorders category) is observed as most significant result for functional annotation filter 2 (P-value = 3.88e − 10). This association does not reach Bonferroni significance in other filter categories implying the relevance of LOF and deleterious variants from this category and their link to alcohol abuse. *ADRA2B* has been linked to diseases such as hypertension, obesity, epilepsy, etc^39–41^ and its association with addiction is also known^42^, but the specific link with alcohol consumption and abuse has not been reported. Notably, the Non-functionally annotated results for this gene and phenotype were very non-significant.

In the functional annotation filter 3 category, we observed an association between the gene *NPR3* with “anxiety behavior” ICD-9 309.24 (diagnosis category “adjustment disorder with anxiety”) with P-value = 1.21e − 09. The result however was statistically non-significant for functional annotation filter 1 and All Variants, underscoring the contribution of LOF variants in filter 3 to these associations. Natriuretic receptors are well known to play essential role in blood pressure regulation. These receptors are also known to be very important in fluid regulation in central nervous system and thus can effect emotional behaviors such as anxiety^43^.

We repeated this analysis using only the more highly significant top 5 clinical laboratory measure associations from each rare-variant filter from the results presented in Fig. 3A. As previously mentioned, the association between *GPT* gene and alanine aminotransferase is the most significant result in all 4 categories consisting of functionally annotated variants. This association is not significant in non-functionally annotated category. Also, it is interesting to note that top 5 associations that are significant in non-functionally annotated category are not significant at all in other categories and do not pass Bonferroni significance in general.

**Figure 4:**
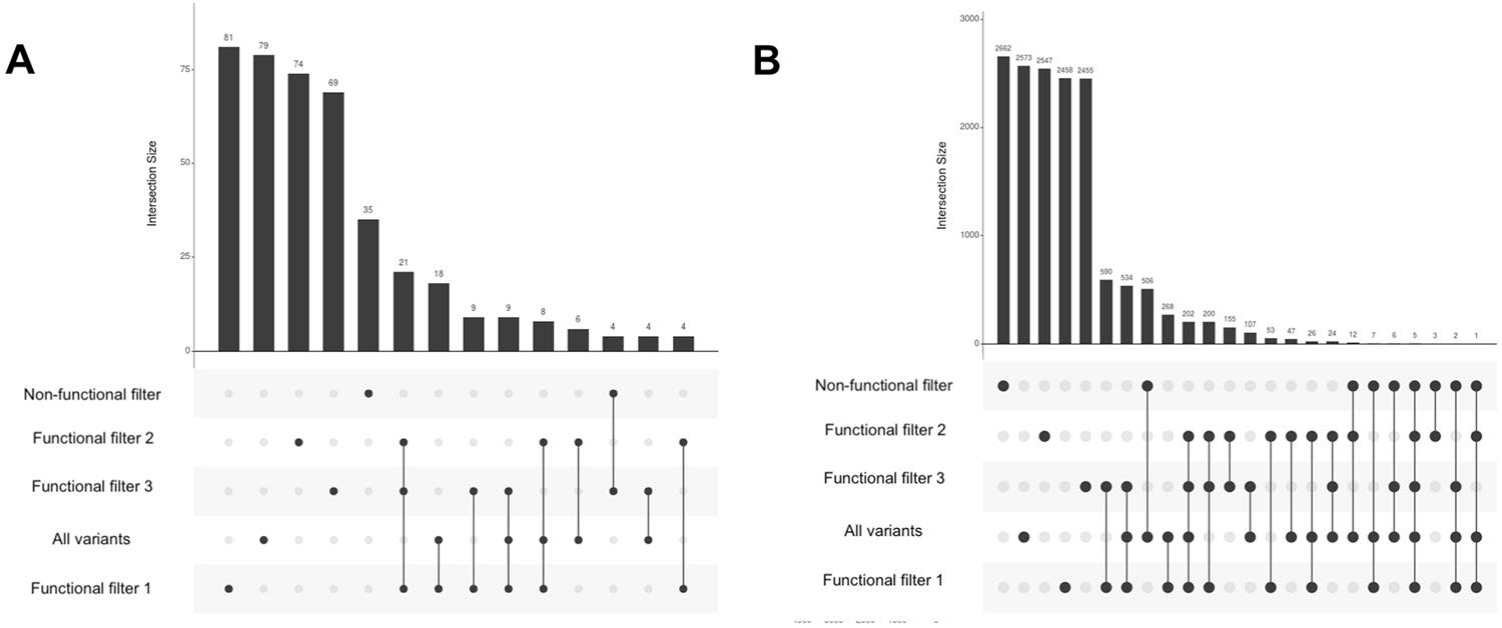
Intersection of results passing a P-value < 0.001 for associations from different rare variant filtering strategies. The results for clinical laboratory measurements are on the left in (**A**) and the results for ICD-9 codes are on the right in (**B**). This figure shows the breakdown of these results based on how the variants were filtered, and whether or not the association was found only with one approach for filtering variants, or more than one way of filtering variants (lines connected between filtering method). The “intersection size” shows for each combination of filtering approach how many results passed the P-value cutoff, and set size indicates across that filtering approach total how many results there were.

Next, we explored the results intersecting among the rare-variant filters and again looked at the count of results with P-value < 0.001, shown in Fig. 4. For clinical laboratory measures, we observed 8 results that were in functionally annotated filtered categories. For ICD-9 diagnoses, we observed only 5 results that were shared among all categories implying that the effect of association is from the combination of all functionally annotated and not annotated variations, 200 results that were only present for functionally annotated filters, and 202 results in both the functionally annotated and all variants filter.

The intersection plots shown in Fig. 4 for the counts of associations shared in each category highlight results due to various functionally annotated filters. There were 155 associations in functional annotation filter 2 and 3 in the ICD-9 based associations and 6 associations for the laboratory measures t indicating that these associations were mostly influenced by LOF and deleterious variants.

#### Associations Only Identified For Functionally Annotated Filters

**Figure 5:**
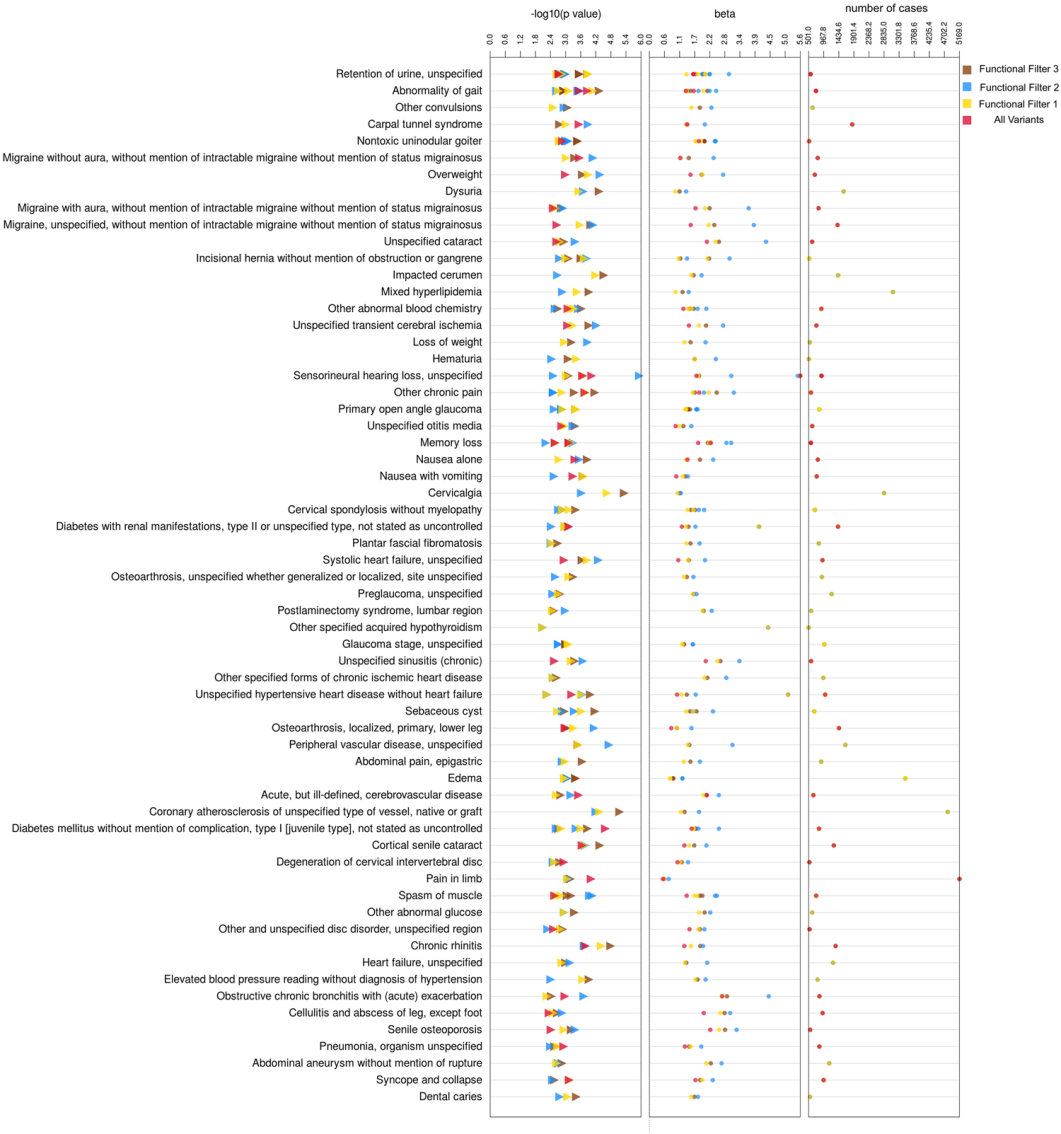
PheWAS-view plot showing P-values, Beta and case number track for all results with strongest associations derived from filtering on functionally annotated variants.

The PheWAS-view plot in Fig. 5 represents the common results identified from all functionally annotated filters, with minimum number of cases 501 (track 3 in Fig. 5). Among the top most significant associations that were found only by functional annotation filtering of variants, we identified associations between the *FOS* gene with “sensorineural hearing loss” ICD-9 389.10 (See Table 1). One factor that causes sensorineural hearing loss is noise and studies in mouse models have suggested that noise exposure activates the MAPK signaling pathway and the *FOS* gene among other genes is up-regulated in MAPK Signaling pathway^44,45^. Another interesting association was observed is between gene *ATF7* and the ICD-9 diagnosis 278.02 (overweight) (see Table 1). Even though this result did not achieve statistical significance, it might reflect clinical importance with further study. The *ATF7* gene is known to be linked to familial atrial fibrillation^46^ but its association with obesity is not completely known.

####  Associations Only Identified for Variants Not Functionally Annotated

We explored the top-most associations where the effect was not due to functionally annotated variants, only due to associations with non-functionally annotated variants. We identified 2120 such associations from the ICD-9 analyses and 31 associations from the clinical lab analyses where the results are filtered at P-value of 0.001. Among the most significant associations is a novel association between the gene *PTGS2* (Prostaglandin-Endoperoxide Synthase 2) and ICD-9 493.20 (chronic obstructive asthma) with P-value = 4.90e − 08 and also between the gene *ADRA1D* and ICD9-9 780.93 (memory loss) with P-value = 7.64e − 07, where the gene *ADRA1D* is already known to be associated with Schizophrenia^47^. All Bonferroni significant results in this category are shown in Supplementary Table 1.

The top most associations are between gene *NGF* and WBC counts (P-value = 5.24e − 06, normalized beta = −4.55) and gene *AZIN2* and creatinine levels (P-value = 5.94e − 06, normalized beta = −4.54). Both of these associations have not been reported previously by rare variant association studies. Nerve Growth Factor *(NGF)* has been known to act in inflammatory responses in rat studies^48,49^ and is also known to be responsible for T and B-cell activation in humans^50^, therefore, its association with WBC counts based on rare variants offer further evidence. Further EHR-based research could help in providing useful insights into understanding the genetics behind these results.

### Associations with diagnoses matching the diagnoses for drugs of the DrugBank database

**Figure 6:**
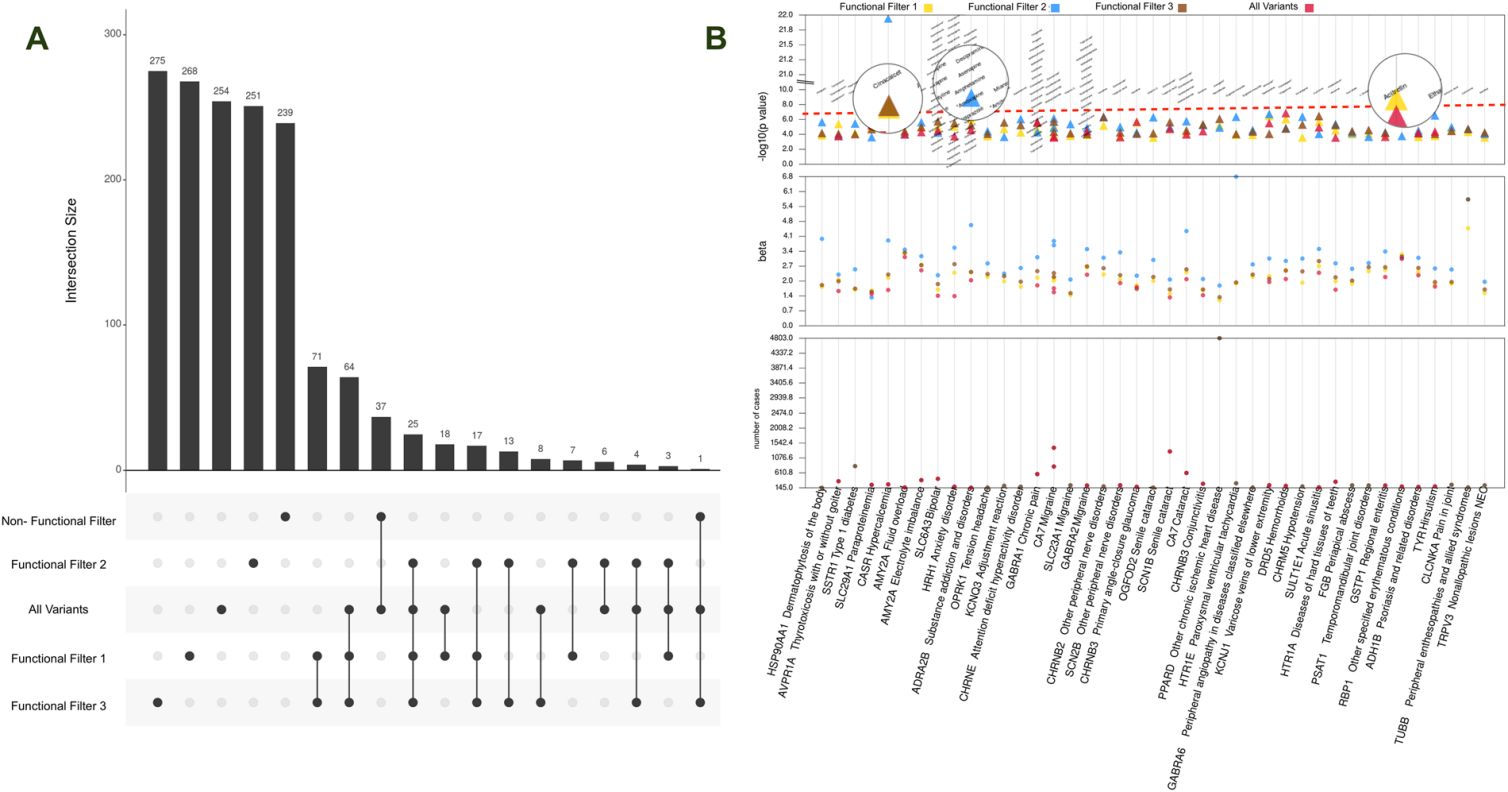
(**A**) Shows the intersection of results where the diagnosis of the association matched the diagnosis used for the drug targeting the gene of the DrugBank database. A total of 874 associations had a match between the target gene, associated phenotype, and the diagnosis that drugs are prescribed to target that gene, for p < 0.001. (**B**) Represents the forty-one associations where there was a match between the target gene, associated phenotype, and the diagnosis drugs are prescribed for that target that gene, for p < 0.001. The x-axis shows the disease description and tracks from top to bottom show -log10 P-value, the magnitude and direction of effect, and the number of cases. Colors represent the different filters applied to the variants before gene based association testing. The Drug name is listed in the same column as the association showing the drug prescribed for the diagnosis listed. Bonferroni significant associations are shown in larger size on the plot.

We characterized gene-disease associations where the diagnosis matches the reason drugs are prescribed that target specific genes. The DrugBank database provides list of genes that are targets for drugs, along with the condition the drugs are prescribed for. We mapped these gene and drug combinations to the ICD-9 code ranges corresponding to disease diagnosis. We matched results below a P-value threshold of 0.001 to the DrugBank listed ICD-9 codes range as explained above. A total of 1,277 associations (7 out of those associations were Bonferroni significant) had a match between the target gene, associated phenotype, and the diagnosis drugs that are prescribed to target that gene. There were 874 unique gene – phenotype combinations. Figure 6A shows how these results across individual ways of filtering rare variants, and when the associations were present with more than one way of filtering rare variants. In supplemental materials, we describe in more detail the number of associations we had depending on the functional annotation of variants.

Two of the top associations, apart from the *CASR* results already described, were for functionally annotated filtered variants. We found an association between *ADRA2B* and “substance addiction and disorders” (ICD-9 305.00; lowest P-value = 3.88e − 10; beta = 4.51; functional annotation filter 2), the drugs known to target this gene include Amoxipine, Amphetamine, Loxapine, Clozapine, and the drugs are prescribed for are depression, stress and attention deficit hyperactivity disorder (ADHD). There were also associations between *RBP1* and ICD-9 695.89 “other specified erythematous conditions” (lowest P-value 1.28e − 09, beta = 3.25, functional annotation filter 1). *RBP1* plays an important role in transportation of vitamin A to epithelial tissue and thus is a gene target for the drug Acitretin^51^ which is used to treat psoriasis, squamous cell carcinoma, chronic hand dermatitis, and malignant melanoma, among others^52^.

In order to further contrast how the significance of results changed depending on variant filter, Fig. 6B shows these results in PheWAS-view plot^53^ where we present P-values, betas and the number of cases for each unique gene-phenotype combination. We also list the drugs as listed in the DrugBank database that are prescribed for the matching diagnosis code. Notably in the figure, regardless of the annotation or all variant filter, for each association the direction of effect is consistently positive. Thus, all associations are with a protective direction of effect for the disease conditions of the associations. In Supplemental file 1 we describe in detail how many associations, and how much overlap, there was for these associations depending on variant filtering.

### Cross-phenotype Associations

Exploring cross-phenotype and potentially pleiotropic associations are possible with PheWAS studies due to a wide range of phenotypes evaluated at once. We filtered all results at P-value threshold of 1.08e − 08 and number of cases greater than 150 (for ICD-9 code associations) to explore cross-phenotype associations. We did not observe any cross-phenotype associations where results were all Bonferroni significant. To explore these results further, for each gene where P-value with phenotype was Bonferroni significant, we also extracted results for same genes at P-value < 1e − 04. Here we only report unique gene-phenotype combinations from all filter categories as represented in Supplementary Fig. 5. This exploratory search of cross-phenotype associations at an exploratory P-value cutoff resulted in several interesting observations. For example, we see association among gene *ABCA1* with HDL levels, total cholesterol levels, and irritable bowel syndrome (IBS). The drug Probucol is used to target *ABCA1* gene which helps in controlling cholesterol and is also known to lower HDL cholesterol levels. In the MyCode dataset, we observed 1,486 patients that are cases for IBS and out of these patients 528 have total cholesterol > 200 mg/dl and 198 patients have HDL <40 mg/dl. A connection between lipid levels and IBS warrants further investigation.

Next, we also observed associations of various phenotypes such as hyposomality (abnormal levels of electrolytes), hypopotassemia, chloride levels and septicemia with the gene Cytochrome oxidase 6 C (*COX6C)*. The only Bonferroni significant association with *COX6C* is abnormal electrolyte levels but this exploratory search points to the direction of the changes in body caused due to sepsis infection. Septicemia can cause abnormal levels of potassium, sodium, chloride, etc^54,55^. Cholic acid is used to target *COX6C* in treatment of adults and children with bile acid synthesis disorders such as Zellweger Syndrome^56^. Cytochromes are known to be crucial during development of sepsis^57,58^ and there is a relationship between cholic acid levels increasing with sepsis.

### Pathway Analyses for Gene-Sets

With the results of our DrugBank PheWAS, we also used gene set enrichment analysis to see if there were multiple genes within the same genetic pathways, that had been associated with the same phenotype. This provides further information about key pathways impacting phenotypic variability, and the impact of perturbing biological networks on outcomes. This approach can also identify multiple potential drug targets across a single pathway. We used the P-values from the regression analyses, separated by each phenotype and variant filtering approach, and ranked results from most significant gene association to least significant association. We then performed gene-set enrichment analysis (GSEA)^59,60^ for the results of each phenotype and filtering approach separately (described further in methods)

Supplementary Fig. 6 shows an overview of number of results in ICD-9 code categories at FDR q-value < 0.25 for each of the filter categories. Similarly, Supplementary Fig. 7 represents an overview of the number of results from clinical laboratory measurements. In our analysis, we did not identify the enrichment of highly-significant genes in any pathways, we instead observed that less significant genes (ranked lower in the list) were enriched in pathways. These results are plotted in a heatmap in Supplementary Fig. 8.

We also explored gene set enrichment analysis at a non-stringent FDR q-value threshold of 0.001. Counts varied from range of 1-5, we picked all gene-sets, gene and phenotype combination where minimum counts of genes are 3. Using a less stringent approach with the association results resulted in combination of 18 genes and 3 gene-sets (GO Drug Metabolic process, KEGG Drug Metabolism Cytochrome P450 and KEGG Retinol Metabolism) that are found to be most enriched in our analysis. These results are shown in Supplementary Fig. 9.

It is not surprising to see drug metabolic processes as the top results from GSEA since we started our analysis with genes that are common drug targets. We observed that in majority of cases for each of these enriched pathways, that even though we performed gene set enrichment separately, there were multiple filter categories (evident from overlaying points in Supplemental Fig. 9) that showed similar gene enrichment results. We did however observe some results where genes were found to be enriched in pathways for results only from one functional variant filtering category. For example, all the genes listed in Supplementary Fig. 9 in the KEGG retinol metabolism pathway are from associations with triglycerides for functional annotation filter 2, there was no enrichment for these genes from other variant filters in this pathway.

## Discussion

Association analyses for rare variants is another strategy in the search for uncovering the hidden heritability of complex diseases^61^. Single variant analyses for rare variants can be under-powered to detect meaningful associations. Thus, collapsing or binning based methods are an important approach to provide enough power for identifying the impact of rare variation on phenotypic variability, these tests can be further refined by filtering variation for functional impact. For this study, we filtered rare variants for each gene in various ways to characterize how much results changed depending on the type of variants chosen.

We identified 91 novel rare variant associations. Many of the results clearly recapitulated the known function of those genes on outcomes, some from common variant association testing, even though the rare variant associations themselves were novel. We also had additional results identifying new hypotheses for gene impact due to rare genetic variation. For example, we observed associations between functionally annotated rare variants in the gene *TACR1* and chronic sinusitis, as well as associations between functionally annotated variants in *ATF7* and obesity related diagnoses (results not passing multiple burden threshold).

One of the unique approaches of this study was to compare and contrast results across different ways of filtering rare variants by function. Gene-based association testing is still relatively new, and annotation of rare variants to identify candidates for study is also a quickly growing and developing field with more and more emerging bioinformatics tools. Our study showed no single filtering approach with superiority over another filtering method. There was variability in the top most significant results for each filtering approach, variability in the number of significant association results for each filtering approach, and our most significant association result also came from the filtering method with the least number of highly significant associations. Thus, our study shows the utility of using different filtering approaches for rare variants when seeking out new genetic associations. For example, our associations between as *CASR* and hypercalcemia, *GPT* and alanine aminotransferease and *UGT1A* genes and bilirubin levels, were identified in all ways of filtering functionally annotated variation. We also tested for associations for rare variants within genes for all variants *except* that are functionally annotated. We observed that in the non-functional annotation filter category, the results were overall less significant when compared to filtering based on annotated variants even though the largest number of associations passing a P-value cutoff of 0.001 were not annotated. With the overall weaker effects of associations when not filtering variants by functionality, we have confirmation of the importance filtering novel variants by functional impact for gene based association testing.

Because DrugBank provides genes that are known drug targets with linked medications that prescribed for specific diagnoses, we linked these diseases to ICD-9 code ranges and identified associations that matched both the gene target and the diseases the specific drugs are used to target. Our analysis resulted in 254 associations at P-value < 0.001 that linked to similar range of ICD-9 codes as diseases for which drugs are prescribed. Again, most of the results passing our P-value cutoff consisted of functionally annotated variants where 5 different algorithms were used to predict LOF, non-synonymous, and deleterious variants. Thus, this underscores again the importance of functional annotation in rare variant association analysis to identify biologically relevant results.

We also explored potential pleiotropic associations, and unsurprisingly we found many cross-phenotype associations for highly correlated phenotypes. However, there were some intriguing cross phenotype associations. For example, Bonferroni significant results showed association of *COX6C* with electrolyte levels but also associations with other phenotypes such as septicemia, chloride and potassium levels for results below P-value 1e − 04. While these phenotypes are interrelated, we may be seeing more of a reflection of the complex interplay between genetics and these phenotypes, not just the correlation between these phenotypes. We also performed pre-ranked gene-set enrichment analysis and we identified 3 pathways and 18 genes that are enriched from low-significant ranked genes from our list. We did not find any of the genes that were highly significant in our associations to be enriched in any pathways.

### Limitations

We adjusted our regression models consistently by age, sex and the first 4 principle components corresponding to genetic ancestry. We could have potentially missed the effect of other covariates on the associations. However, from one phenotype to another the most relevant covariates can vary, and we performed high-throughput associations over hundreds of phenotypes. This is regularly a limitation in PheWAS when performing high throughput associations. The benefit of PheWAS is that the results generate new hypothesis for further research, where any individual association models can be investigated in the future in a more comprehensive manner, including more phenotypic development and exploring various covariates and their impact on the model.

## Conclusions

In this manuscript, we have presented a gene based PheWAS-study by collapsing functionally annotated and non-annotated rare variants (MAF < 1%) into gene bins that are known drug targets. We used a burden based approach to highlight the direction of effect for associations for the genes tested. We have presented several associations where our results clearly reflect the known function of these genes, underscoring how changes to the proteins through rare variation impact phenotypic variation. We also found associations where there is less of a known relationship between gene products and phenotypic variability, which could lead to new hypotheses for further research. Our analyses highlight the importance of filtering rare variants by functional impact before testing associations. We identified interesting cross-phenotype associations. Our future work includes more refined phenotyping of variables identified in the associations of this study and also developing high throughput technique to adjust the models for related phenotypes. We will also further explore the potentially pleiotropic results of this study.

## Methods

**Figure 7:**
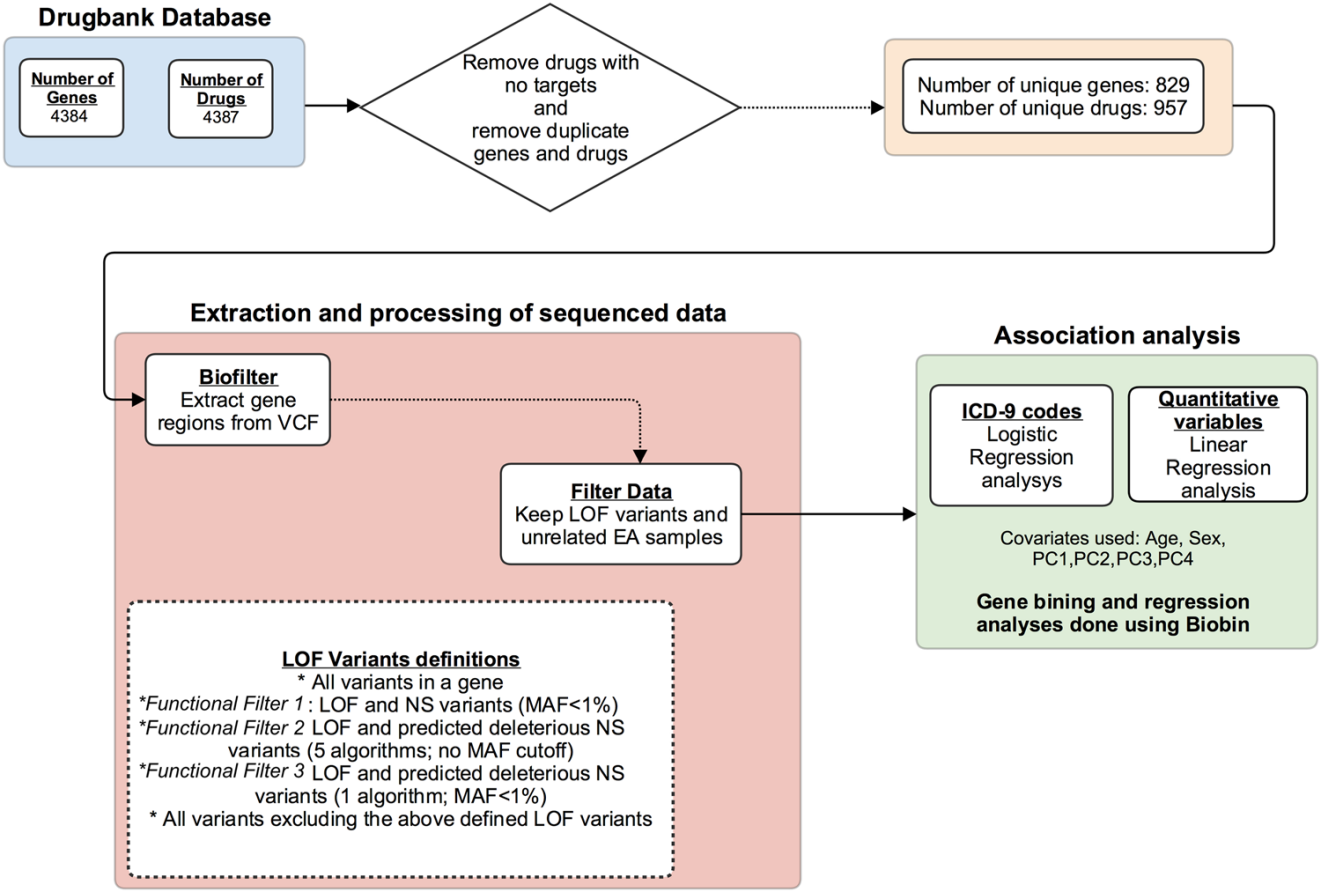
Flow chart of the analyses of this study.

Figure 7 provides an overview of the sequence of steps and tools that were used for the analyses of this manuscript.

### Extraction of Genes from DrugBank Database

The DrugBank database^19^ contains 4,387 different genes and drugs. A drug can have multiple target genes or a single gene can also be the target of multiple drugs. A total of 3 drugs (Captopril, Fluorescein and Glycine) did not have target genes in DrugBank. We obtained unique list of 829 genes and 957 drugs after removing duplicates.

We also retrieved chromosome and base pair locations for these 829 unique DrugBank genes in the latest genome assembly build 38 using Biofilter version 2.4^62^ and the (Library of Knowledge Integration) LOKI database version 2.2. Of the list of 829 genes, 19 gene identifiers, listed in Supplementary Table 3, were unrecognized by the Biofilter software. For these genes, alternate identifiers were searched for using NCBI PubMed (https://www.ncbi.nlm.nih.gov/pubmed) and Gene Cards^63^ database. Table 2 lists the genes unrecognized by Biofilter as well as the alternate gene recognized by Biofilter. For the genes, the build 38 regions were retrieved the same way as explained above. Further, for our final gene list, we only considered genes in autosomal regions, and excluded mitochondrial genes.

**Table 2:** Alternate Gene Identifiers for Genes Unrecognized by Biofilter.

Unmatched Gene Symbol	Alternate Gene Symbol
*ABP1*	*AOC1, DBNL*
*ACCN1*	*ASIC2*
*ACCN2*	*ASIC1*
*ADC*	*AZIN2, GADL1*
*CCBL2*	*KYAT3*
*CDO-1*	*CDO1*
*DKFZp686P18130*	*FECH*
*GAD65*	*GAD2*
*GIG18*	*GOT1, GLCCI1*
*GPR44*	*PTGDR2*
*LEPRE1*	*P3H1*
*LEPREL1*	*P3H2*
*LEPREL2*	*P3H3*
*PGCP*	*CPQ*
*TUBB2C*	*TUBB4B*

Due to the unspecific and changing nature of gene symbols, gene annotation sources such as Entrez (the source of gene symbols and locations used by Biofilter) can have gene symbols annotated with multiple genic regions. In the current dataset, there were 5 genes with multiple regions listed in Table 3. We tested variants in these multiple regions even though they were mapped to same gene name.

**Table 3:** Genes Annotated with Multiple Regions by Biofilter.

Chr	Gene Name	Start Position	End Position
12	*C1R*	7080209	7082108
12	*C1R*	7085860	7092447
5	*CDO1*	115804733	115816954
5	*CDO1*	115813620	115816111
1	*CHRM3*	239386518	239387227
1	*CHRM3*	239386565	239911462
3	*LTF*	46436005	46465142
3	*LTF*	46468135	46485234
3	*RARB*	24829344	25120621
3	*RARB*	25174332	25597932

The variant calling pipeline for the samples of this study was using the build 37 genome assembly, thus the regions for these genes in build 38 were converted to build 37 using LiftOver (Lift Genome Annotations) available as part of UCSC genome browser^64^. For 3 genes: *C1R*, *FCGR1B* and *MUC2*, LiftOver failed to convert regions. These regions were split into multiple regions on same chromosome based on the gene boundaries in the build 37 genome assembly as suggested by LiftOver. Excluding these genes resulted in the final number of 797 unique gene regions (including the alternate gene regions as explained above). In Supplementary Table 2, we provide the list of all these 797 gene symbols and chromosome and base pair locations.

### Extraction of Genes From Exome-Sequenced Data

We had a sample size of 38,568 for this study. Table 4 shows the demographics of our samples. We included all variants in autosomes that passed the Variant Quality Score Recalibration (VQSR)^65–67^ sensitivity threshold of 99.5% for SNPs and 99% for INDELs as recommended in GATK best practices^67^. In our exome sequencing pipeline, we did not call mitochondrial genes. Hence, they were excluded from this analysis. We also filtered the sequencing data to include only unrelated European Americans using genetically informed ancestry estimated via principal components who were >18 years of age. From this QC’ed version of dataset, using GATK we then extracted regions as specified in Supplementary Table 2 to obtain all variants in 797 DrugBank genes further used for association testing.

**Table 4:** Demographic information for the samples of this study after quality control.

		Total 38568	females 22428	males 16116
Age	Min	18.01	18.01	18.12
	Median	62.18	58.71	65.7
	Mean	60.21	57.32	64.22
	Max	88.55	88.55	88.55
BMI	Min	13.18	13.18	13.51
	Median	30.36	30.73	30.04
	Mean	31.64	31.98	31.17
	Max	113.19	85.79	113.19

For our association testing, we used a collapsing approach and binned all variants with minor allele frequency (MAF) <1% within the genes using Biobin^68^. We also binned variants with MAF <1% using various filtering approaches described below:

- **All Variants:** All variants in the DrugBank genes (no filtering based on functionality)
- **Functional annotation filter 1:** Loss of Function (LOF) and non-synonymous variants using SNPEff software^16^ (see the definition of LOF and non-synonymous below).
- **Functional annotation filter 2:** LOF and predicted deleterious non-synonymous variants by using five different predictive algorithms. A variant was predicted deleterious if a consensus of the 5 algorithms listed below indicated that the variant is deleterious. If multiple annotations existed for a given variant (as would be in the case of multiallelic variants or variant annotations specific to a transcript), a variant was considered to be LOF or non-synonymous if ANY annotation for that variant met our specification for LOF or non-synonymous. The following 5 scoring algorithms were used in dbNSFP^69^ to predict the deleterious variants (limited to only SNPs/SNVs):
  1. SIFT^70^ 5.2.2
  2. PolyPhen2 (HDIV training set)^71^
  3. PolyPhen2 (HVAR training set)^71^
  4. LRT^72^
  5. MutationTaster^73^.
- **Functional annotation filter 3:** LOF and predicted deleterious non-synonymous variants by using one predictive algorithm (SIFT). SIFT is one of the most established and commonly used annotation filters, thus we created a less stringent filtering than annotation filter 2 by focusing on a single annotation filter. Thus, we expanded the number of variants evaluated, while still filtering on LOF and non-synonymous variants, using a very established algorithm.
- **Non-functionally annotated variants:** Variants without functional annotation, i.e. the variants that were not included in 2, 3 or 4 above
  These are the definitions of our LOF and non-synonymous variants
- LOF: A variant that has one of the following roles:
  1. Chromosome_number_variation
  2. Exon_loss_variant
  3. Frameshift_variant
  4. Stop_gained
  5. Stop_lost
  6. Start_lost
  7. Splice_acceptor_variant
  8. Splice_donor_variant
  9. Rare_amino_acid_variant
  10. Transcript_ablation
  11. Disruptive_inframe_insertion
  12. Disruptive_inframe_deletion.
*Note that this consisted of all SNPEff roles with a HIGH impact modifier, plus the addition of the disruptive insertion/deletion*
- Non-synonymous Variants: Variants identified as a LOF variant above, or had one of the following roles:
  1. Missense_variant
  2. Inframe_insertion
  3. Inframe_deletion
  4. 5_prime_UTR_truncation
  5. 3_prime_UTR_truncatisplice_region_variant
  6. Splice_branch_variant
  7. Coding_sequence_variant
  8. Regulatory_region_ablation
  9. TFBS_ablation
  10. 5_prime_UTR_premature_start_codon_gain_variant
  11. Non-canonical_start_codon.

### Phenotype Data Extraction From EHR

We extracted international classification of disease version 9 (ICD-9) codes from the electronic health record (EHR) of GHS. For the ICD-9 based data, we created case/control diagnoses, requiring an individual to have 3 or more instances of an ICD-9 code to be considered a case, individuals with less than three but greater than zero instances were dropped out of the analyses in order to avoid including samples with misdiagnosis. Zero instances of an ICD-9 code resulted in the individual being considered a control. As a result, we had a total of 541 case/control based diagnoses used in our association testing.

A total of 35 clinical lab measures were also extracted from the EHR; we used the median lab value measured from the longitudinal data for each individual. Some individuals had more clinical lab measures than others, we used the median to obtain a general reflection of individual clinical lab measures. In previous publications^11–13^, we have shown the efficacy of these measures in association studies.

We previously identified that quality control and transformation of clinical laboratory measurements was needed to meet all assumptions for the statistical tests of association^11^. Units of measurements are different at various GHS laboratories and devices used at the time of care thus we standardized these observations following Logical Observation Identifiers Names and Codes (LOINC) guidelines. We did not include any measurements where the units reported were different than LOINC units and/or if the conversion was not possible. We excluded outliers where measurements were not within + −3 standard deviations. Median values were calculated for each patient using all their measurements from the EHR available as outpatients. Values were also transformed to obtain normal distributions. Table 5 lists all the clinical variables used for the analysis and their respective transformation methods applied if necessary (left blank if not required).

**Table 5:** Clinical lab phenotypic variables tested for PheWAS.

	Clinical Lab Trait	Transformation
1	Alanine aminotransferase - serum plasma	Natural Log
2	Albumin - serum plasma	Natural Log
3	Alkaline phosphatase - serum plasma	Natural Log
4	Anion GAP - serum plasma	—
5	Aspartate aminotransferase (AST) - serum plasma	Natural Log
6	Bilirubin - serum plasma 0.001	Natural Log
7	Calcium (Ca) - serum plasma	—
8	CARBON_DIOXIDE_CO2_SERUM_PLASMA	—
9	Chloride (Cl) - serum plasma	—
10	Creatinine (eGFR) - serum plasma	Natural Log
11	Erythrocyte Distribution Width (RDW) - blood	Natural Log
12	Hematocrit (HCT) - blood	—
13	Hemoglobin - blood	—
14	Mean corpuscular hemoglobin concentration (MCHC) - blood	—
15	Mean corpuscular hemoglobin (MCH) - blood	—
16	Mean corpuscular volume (MCV) - blood	—
17	Platelet blood count	—
18	Platelet mean volume (MPV) - blood	—
19	Potassium (K) - serum plasma	—
20	Protein - serum plasma	—
21	Red Blood Cell (RBC) count - blood	—
22	Sodium (Na) - serum plasma	—
23	Urea Nitrogen - serum plasma	—
24	White Blood Cell (WBC) count - blood 0.001	Log
25	Fasting Blood Glucose (FBG)	Boxcox
26	Hemoglobin A1C (HBA1C)	Boxcox
27	Cholesterol	Natural Log
28	Free T3	Natural Log
29	Free T4	Natural Log
30	Bicarbonate (HCO3)	Natural Log
31	High Density Lipoprotein (HDL)	Natural Log
32	Insulin-like growth factor (IGF1)	Natural Log
33	Low density lipoprotein (LDL)	Natural Log
34	Triglycerides (TRIG)	Natural Log
35	Thyroid stimulating hormone (TSH)	Natural Log

### Burden-test Analysis

After collapsing rare variants across the 797 genes separately for all 5 types of filtering rare variants, as described above we then used regression to evaluate associations with our 576 phenotypes listed in Supplementary Table 3. The rare variant burden calculated for each individual included weighting based on rarity of the variants, using weighted sum collapsing approach as suggested by Madsen and Browning^74^. Weighted sum collapsing approach to give more weights to rare variants due to their stronger effect sizes is implemented in Biobin. For associations with ICD- 9 diagnoses, logistic regression was used, and for quantitative clinical lab measures linear regression was used. All models were adjusted by the covariates of age, sex and first 4 principal components for ancestry. Below are the regression models for both disease diagnosis analysis (logistic regression) and laboratory measurement analysis (linear regression). Biobin currently does not provide direction of effect from regression analysis. Thus, for each analysis we also calculated direction of effect (beta) using PLATO (http://ritchielab.psu.edu/software/plato-download).

1 $$\begin{array}{c}{Y}_{Disease}={\beta }_{0}+{\beta }_{1}{X}_{BinValue}+{\beta }_{2}Age+{\beta }_{3}Sex+{\beta }_{4}PC1\\ \,\,\,\,\,+\,{\beta }_{5}PC2+{\beta }_{6}PC3+{\beta }_{7}PC4\end{array}$$

2 $$\begin{array}{c}{Y}_{Value}={\beta }_{0}+{\beta }_{1}{X}_{BinValue}+{\beta }_{2}Age+{\beta }_{3}Sex+{\beta }_{4}PC1+{\beta }_{5}PC2\\ \,\,\,\,\,\,+\,{\beta }_{6}PC3+{\beta }_{7}PC4+\,\varepsilon \end{array}$$

In equations (1) and (2), $Y$ refers to the dependent variable ($YDisease$ is binary phenotype trait and $Y{Value}$ refers to quantitative phenotype value), $XBinValue$ refers to the contribution of individual to a gene bin, ${\beta }_{0}$ is the beta coefficent of the model and $\varepsilon$ is the error term.

Associations using different filters for binning approaches (filters as described above) as well as ICD-9 codes and clinical laboratory measures were run separately and then results were combined. All total, considering both case/control diagnoses and quantitative clinical lab measures, we performed 459,072 tests. This resulted in a Bonferroni Correction of 1.08e − 07 using an alpha of 0.05.

### Gene-set Enrichment Analysis

Using the P-values from the regression analyses, separated by each phenotype and variant filtering approach, we ranked results from most significant gene association to least significant association. We then performed gene-set enrichment analysis (GSEA)^59,60^ for the results of each phenotype and filtering approach separately. We ran GSEA using the following gene-set databases:

1. KEGG Pathway
2. GO Biological Processes
3. Immunological signatures
4. microRNA targets
5. transcription factor targets

We ran the analysis using GSEA command line option for each phenotype and then compiled all results for ICD-9 and quantitative variables at FDR q-value < 0.25 into two sets of results to evaluate. For GSEA, we used default options of 1000 permutations for pre-ranked analysis where ranking of genes is based on the significance of P-value obtained from regression analysis. We then explored results from diagnosis codes and laboratory measurements to identify most significant gene-set terms and genes enriched for the phenotypes evaluated.

## Electronic supplementary material

Supplementary Information

## Data Availability

Additional information for reproducing the results described in the article is available upon reasonable request and subject to a data use agreement.
