# Author Correction: Rare variants in drug target genes contributing to complex diseases, phenome-wide

**DOI:** 10.1038/s41598-018-27936-7

**Published:** 2018-10-23

**Authors:** Shefali Setia Verma, Navya Josyula, Anurag Verma, Xinyuan Zhang, Yogasudha Veturi, Frederick E. Dewey, Dustin N. Hartzel, Daniel R. Lavage, Joe Leader, Marylyn D. Ritchie, Sarah A. Pendergrass

**Affiliations:** 1 Perelman School of Medicine, Department of Genetics, University of Pennsylvania, Philadelphia, PA 19104 USA; 2 Biomedical and Translational Informatics Institute, Geisinger, Danville, PA 17221 USA; 3 Phenomic Analytics and Clinical Data Core, Geisinger, Danville, PA USA; 4 Regeneron Genetics Center, Tarrytown, NY 10591 USA

Correction to: *Scientific Reports* 10.1038/s41598-018-22834-4, published online 15 March 2018

The Acknowledgements section in this Article was omitted. The Acknowledgements section should read:

“This project was funded in part under a grant with the Pennsylvania Department of Health (#SAP 4100070267). The Department specifically disclaims responsibility for any analyses, interpretations or conclusions.”

