# Supplementary material for: Rare variants in drug target genes contributing to complex diseases, phenome-wide
Source: Sci Rep. 2018 Mar 15;8:4624. doi: 10.1038/s41598-018-22834-4 (PMC5854600; doi:10.1038/s41598-018-22834-4)
Supplement: Supplementary file 1 — Supplementary Information [file 41598_2018_22834_MOESM1_ESM.pdf]

Rare variants in drug target genes contributing to complex diseases, phenome-wide

Shefali Setia Verma[1], Navya Josyula[2], Anurag Verma[1], Xinyuan Zhang[2], Yogasudha Veturi[1], Frederick E. Dewey[4], Dustin N. Hartzel[3], Daniel R. Lavage[3], Joe Leader[2,3], Marylyn D. Ritchie[1], Sarah A. Pendergrass[2*]

Supplementary Text:

Additional Results:

Over 200 results were observed in each filter category. There were 25 results that showed Bonferroni significant associations with All Variants as well as with all functionally annotated filters. A total of 17 results were observed only in variants filtered by functional annotation before burden testing, not in all variants nor non- functionally annotated filtered variant based associations, highlighting the importance of functional annotation of specific variants in the associations. We observed 13 associations shared among functional annotation Filter 2 and 3, and 8 associations for functional annotation filter 2 and 3 only, which suggests the importance of the specific type of annotation filtering for those associations. The least overlap in various filtering approaches was between the non-functionally annotated -filtered data (no low frequency variants with putative functional impact) and the other filtering approaches. There were 37 gene-phenotype associations (among those with P-value< 0.001) in both the non-functionally annotated filtered data and 'all variants'. There was one association shared across the non-functionally annotated filter based associations as well as functional annotation filter 3.

Next, we further inspected associations that were unique to each filtering approach. **Table** below provides the unique count of associations that are observed exclusively in each filtering category. We noted that among the annotation filtering approaches, the most significant as well as unique associations were observed in functional annotation filter 2 category (176).

Table: Number of associations observed uniquely for each variant filtering approach, where results were not significant with P-value < 0.001 in any other categories, shown with ascending order of count.

Filter Category	Count
Non-functionally annotated Variants	201
Functional Annotation Filter 2	176
All Variants	88
Functional Annotation Filter 3	72
Functional Annotation Filter 1	63

Data Visualization

Plot 2 and Supplementary figures 1, 2 and 3 are generated using circos perl package[75].

Plots 10 and 12 are generated using UpSetR package in Shiny[76].

Plots 11 and 13 are generated using Synthesis- View [51]

Plot 14 are generated using Phenogram package [77]

Supplementary Figures:

Supplementary Figure 1: Circos plot for Functional Filter 2 category

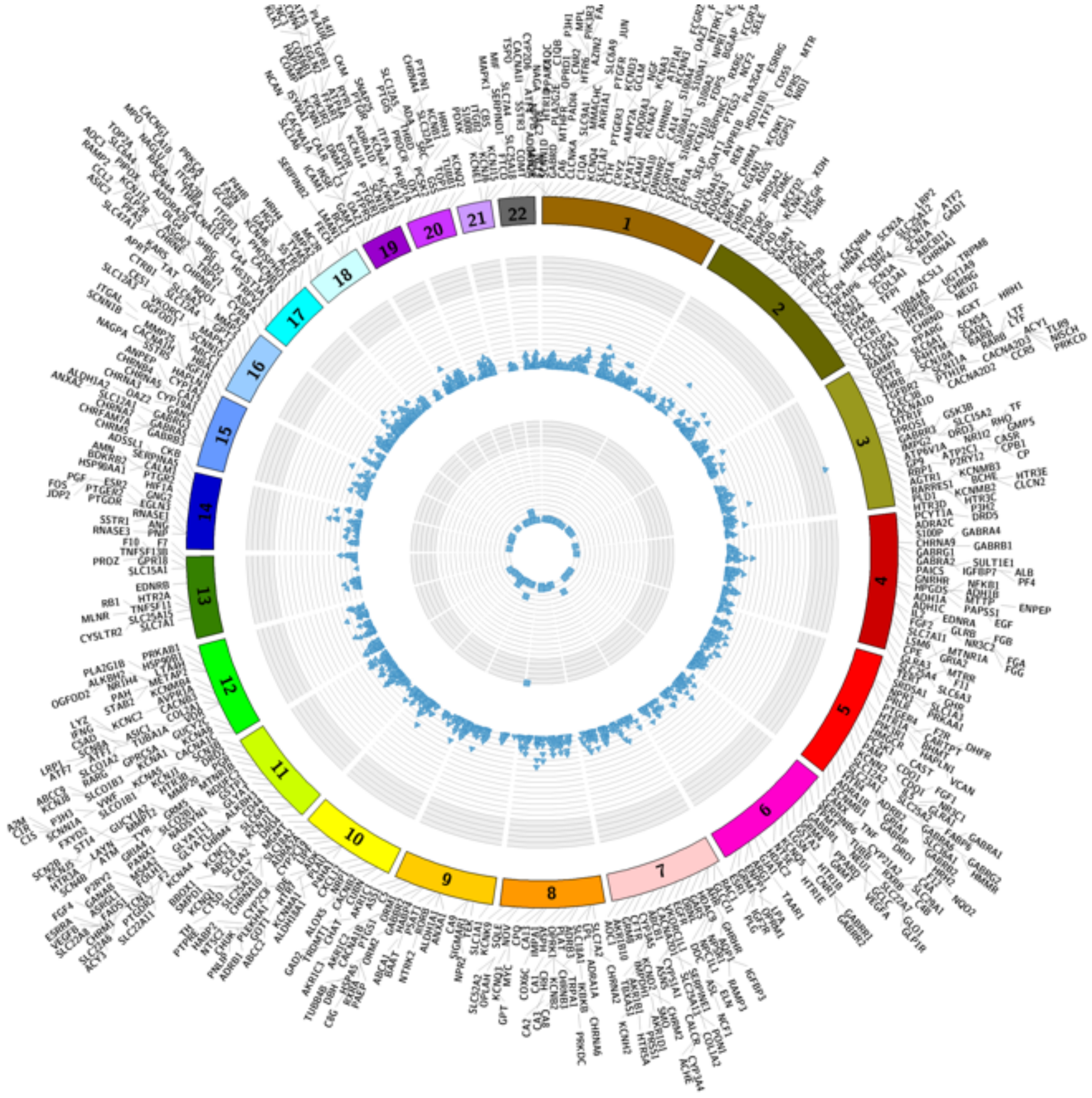

Supplementary Figure 2: Circos plot for Functional filter 3 category

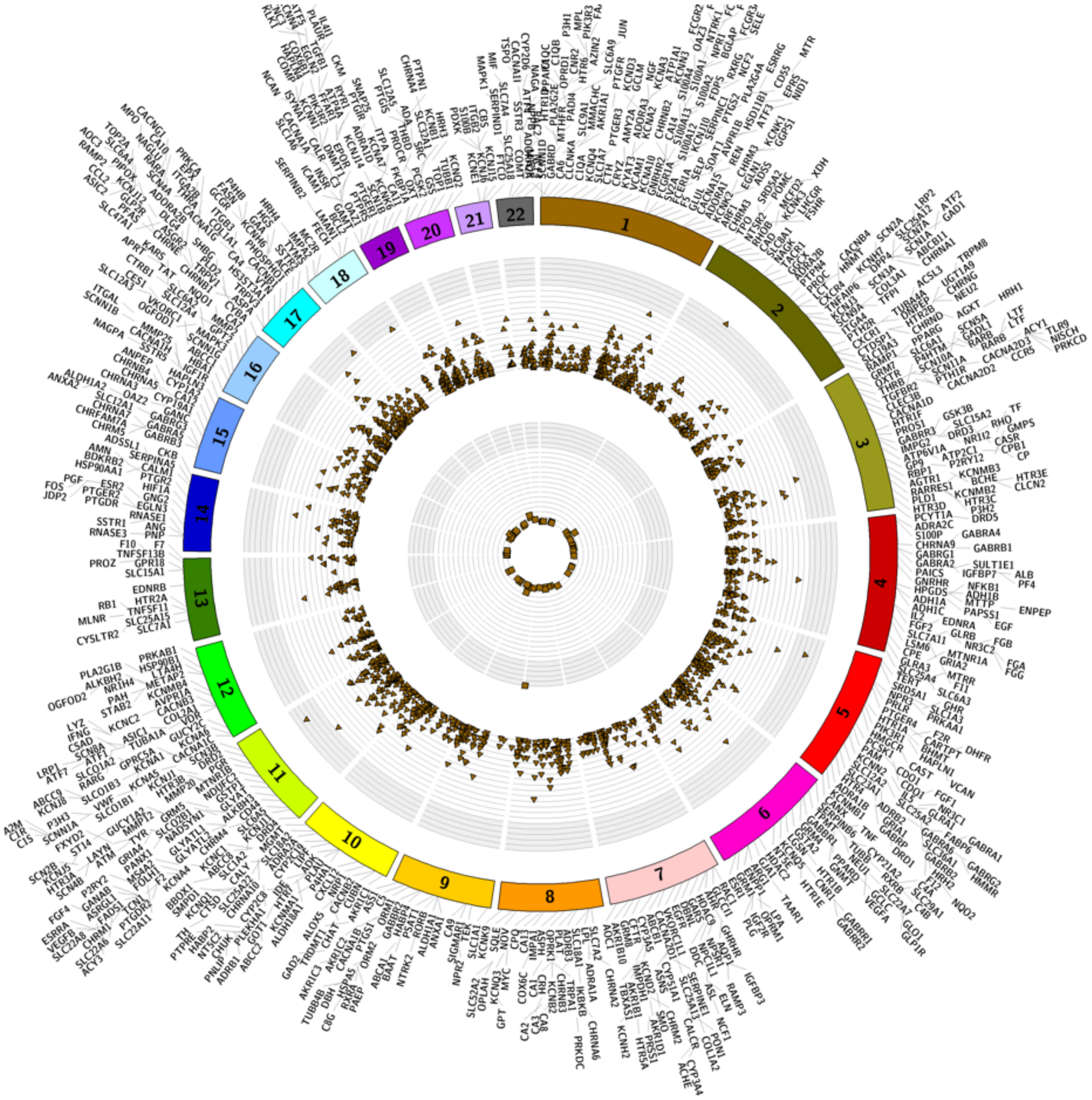

Supplementary Figure 3: Circos plot for All variants category

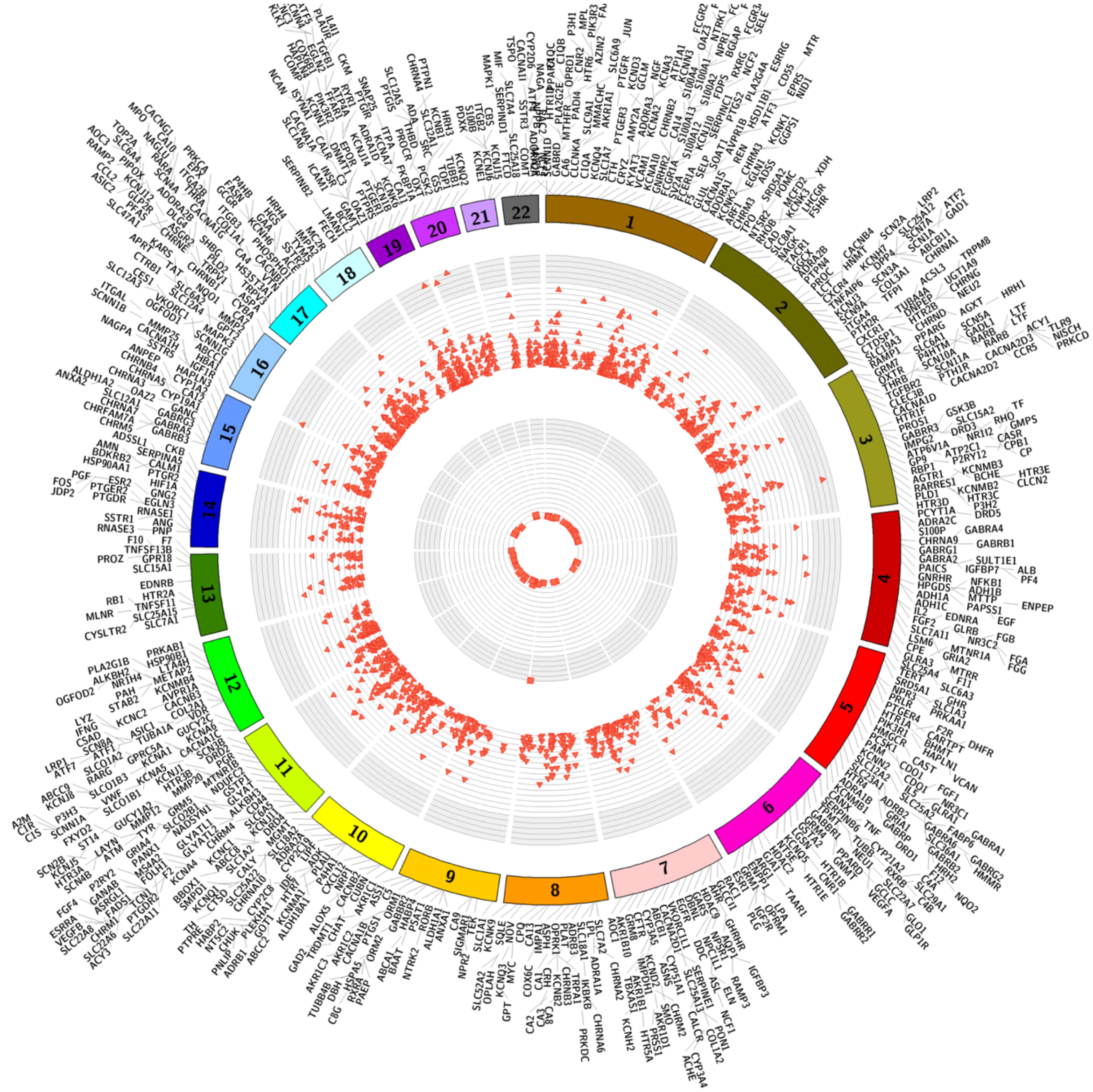

Supplementary Figure 4: Circos plot for Non-functional Filter category

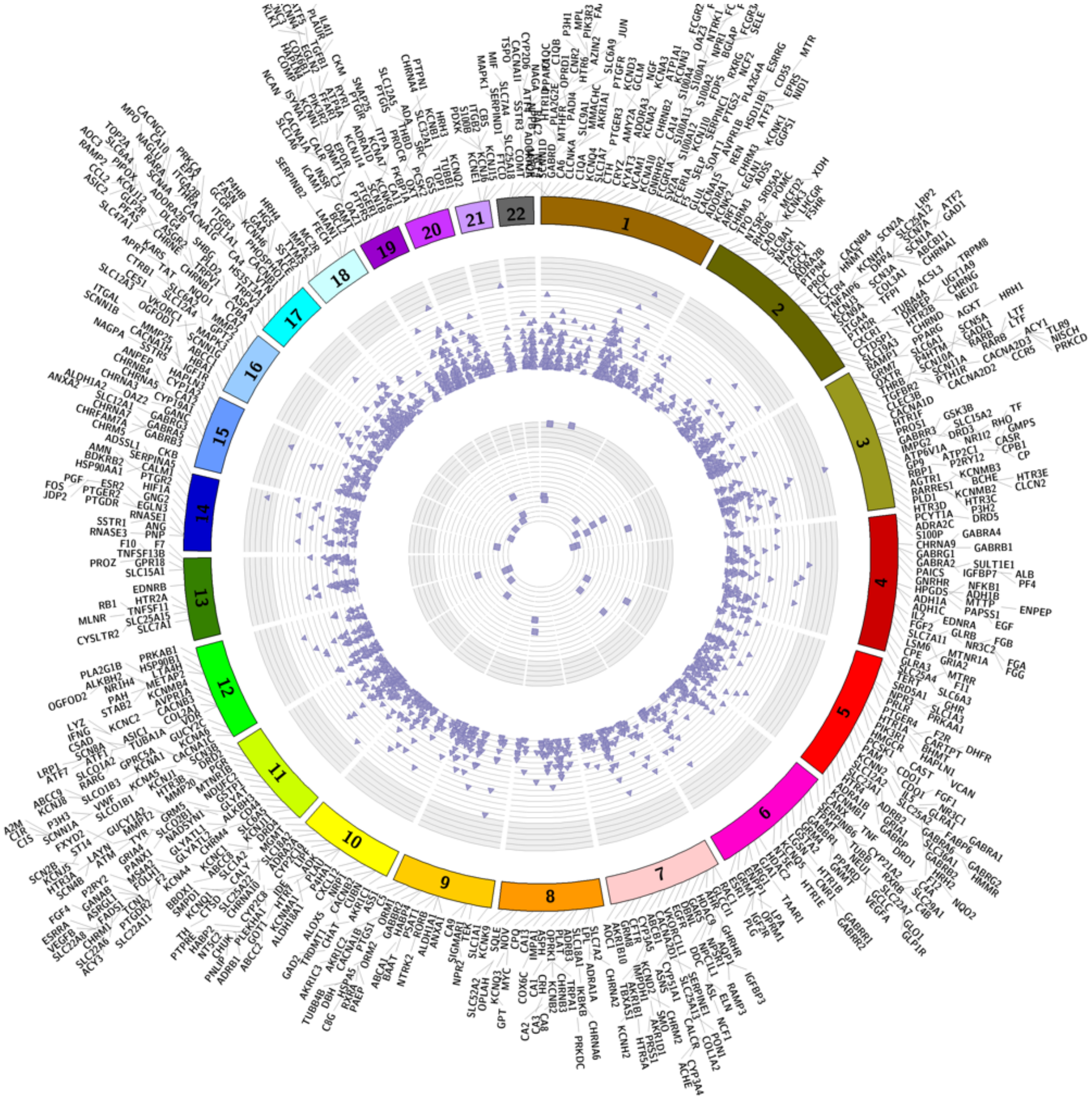

Supplementary Figure 5: Phenogram plot representing cross-phenotype associations from results with P-values < 1e-08. Shapes correspond to ICD-9 code description or Laboratory measurements for both results that are Bonferroni significant and P-value<1e-04 significant

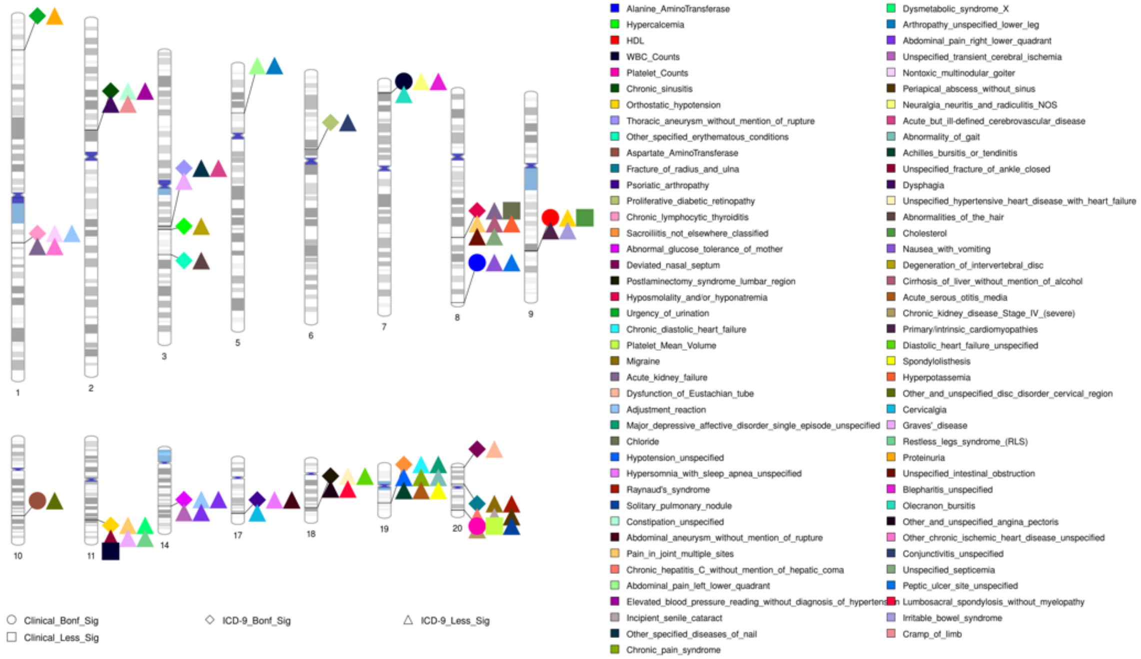

Supplementary Figure 6: Count of results below FDR q-value 0.25 from GSEA for ICD-9 codes

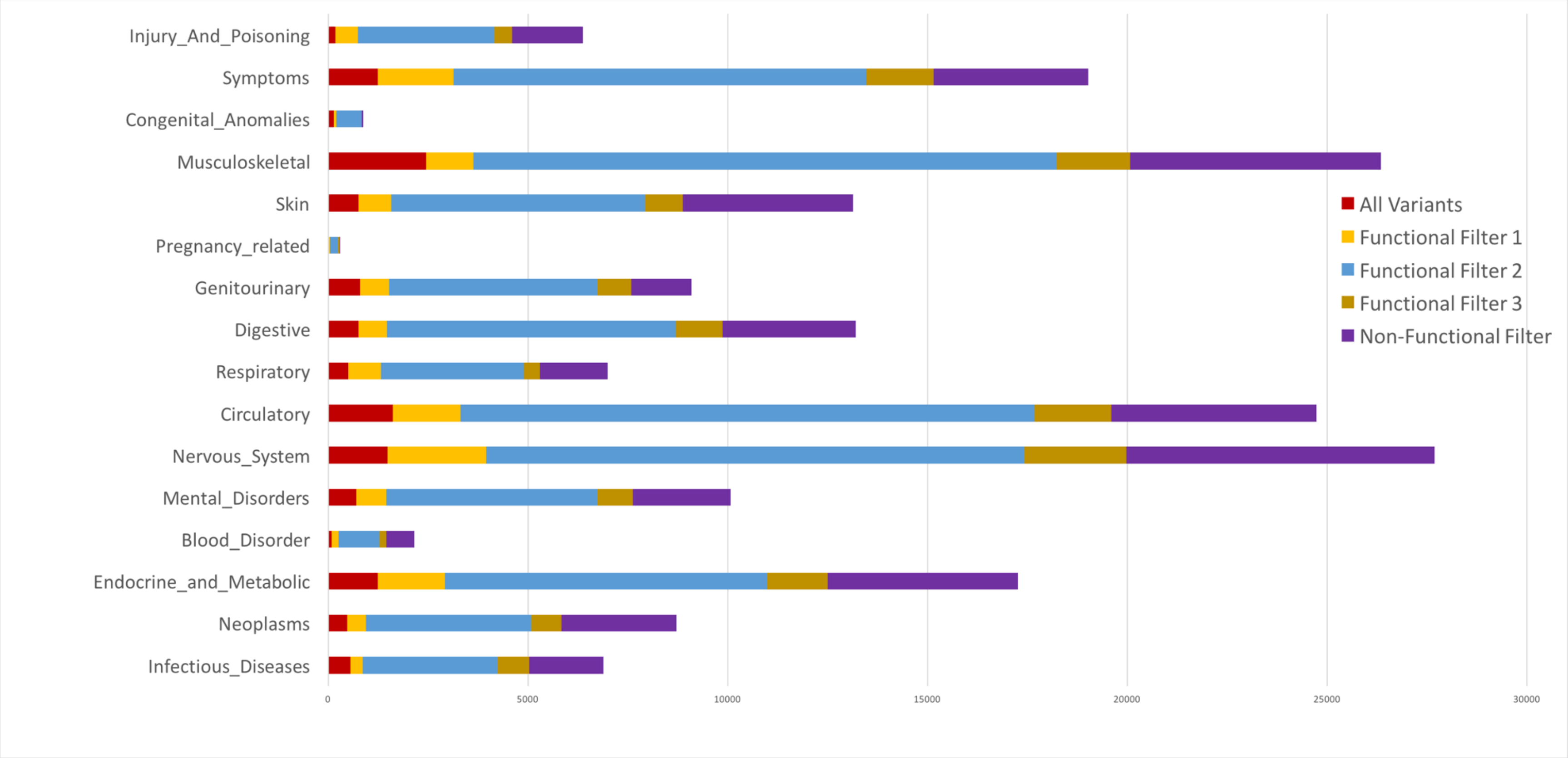

Supplementary Figure 7: Count of results below FDR q-value 0,25 from GSEA for Clinical laboratory measurements

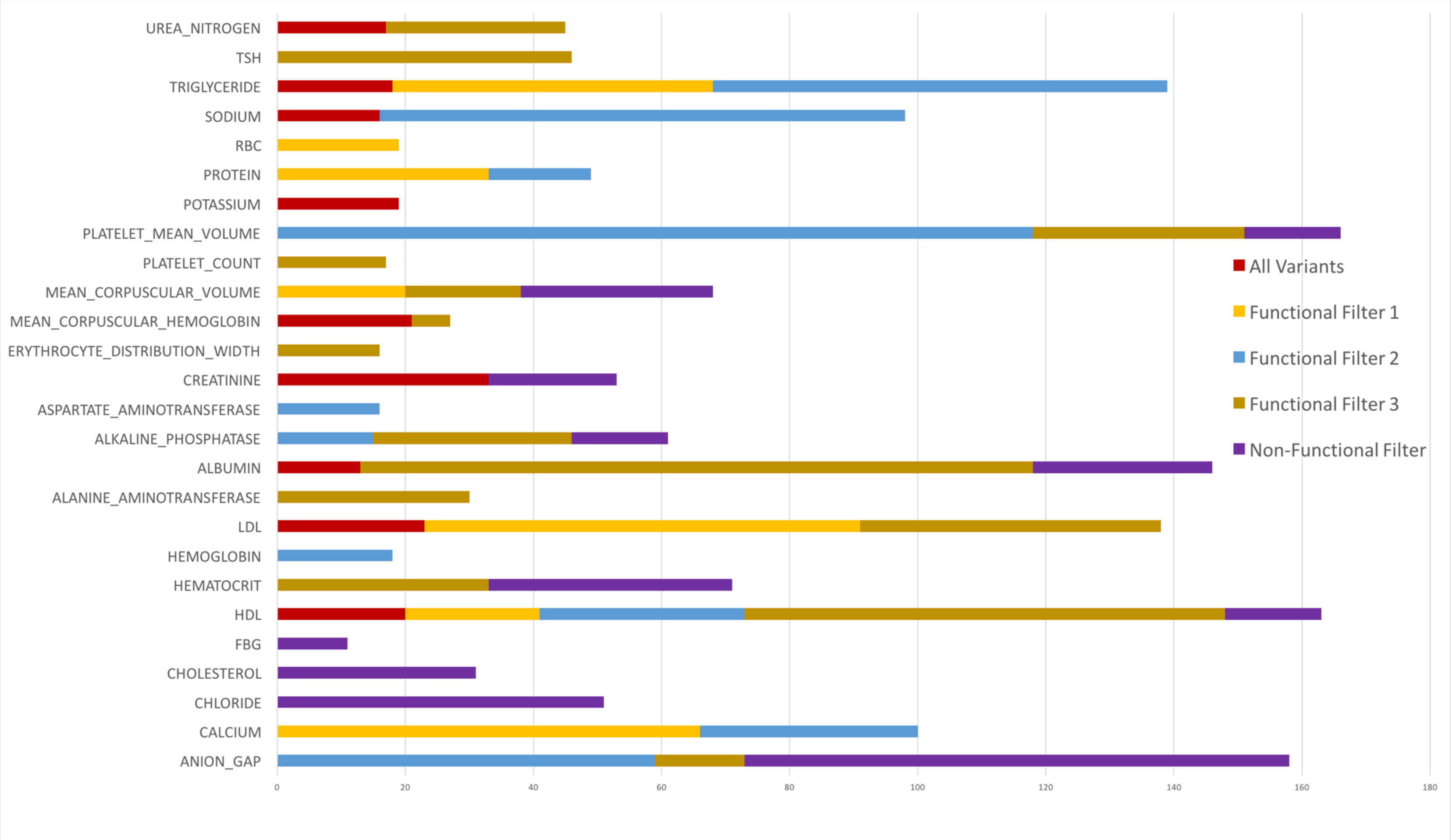

Supplementary Figure 8: Heat map of GSEA results where FDR q-value=0. These results are separated by LOF filter category. X-axis is the gene name and Y-axis represent combination on Phenotype (on right) and Gene-set (On left). Gradient of color corresponds to the normalized enrichment score (NES).

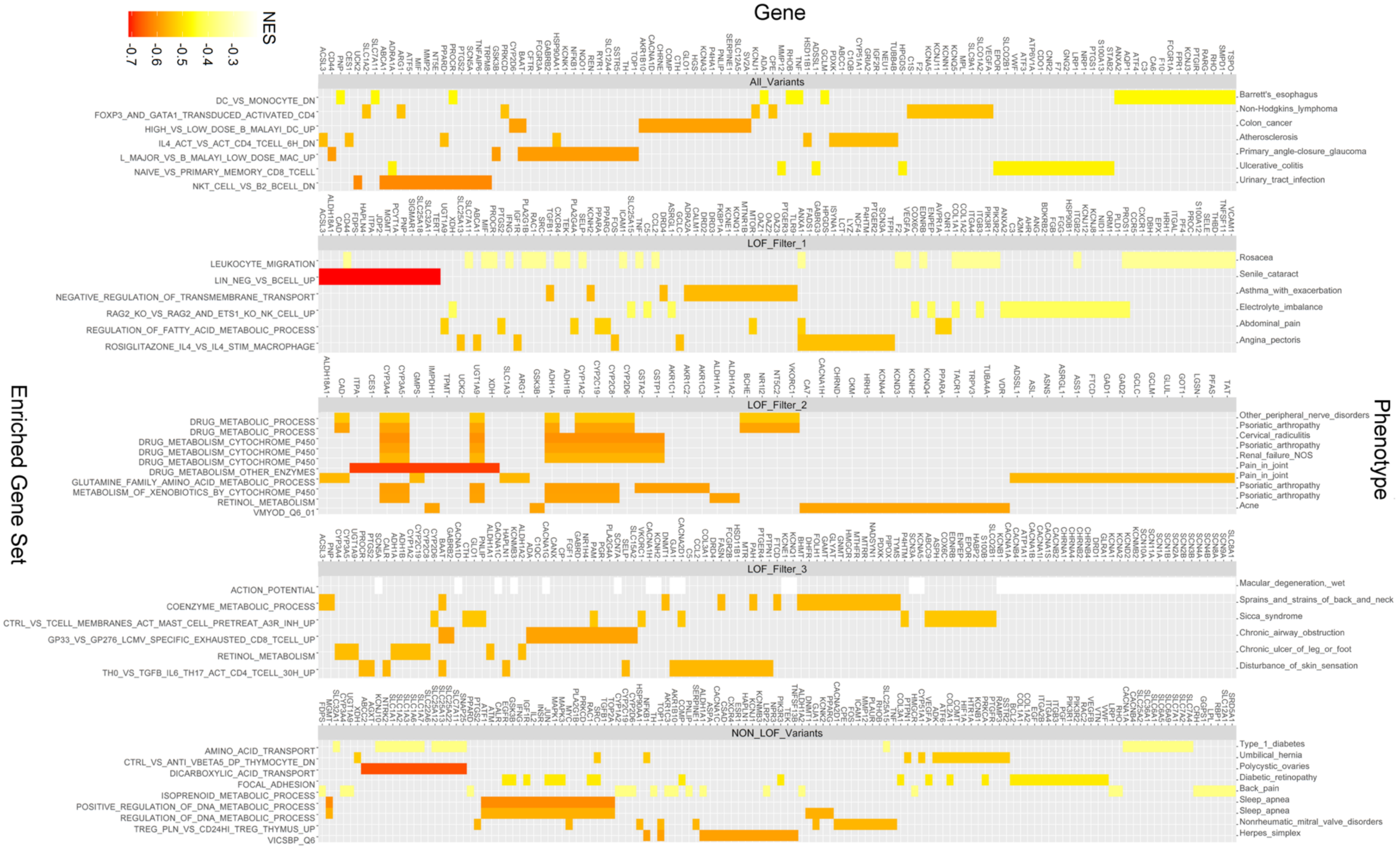

Gene
NES
-0.7
-0.6
-0.5
-0.4
-0.3
Enriched Gene Set
Phenotype
All_Variants
DC_VS_MONOCYTE_DN
FOXP3_AND_GATA1_TRANSDUCED_ACTIVATED_CD4
HIGH_VS_LOW_DOSE_B_MALAYI_DC_UP
IL4_ACT_VS_ACT_CD4_TCELL_6H_DN
L_MAJOR_VS_B_MALAYI_LOW_DOSE_MAC_UP
NAIVE_VS_PRIMARY_MEMORY_CD8_TCELL
NKT_CELL_VS_B2_BCELL_DN
Barrett's_esophagus
Non-Hodgkins_lymphoma
Colon_cancer
Atherosclerosis
Primary_angle-closure_glaucoma
Ulcerative_colitis
Urinary_tract_infection
LOF_Filter_1
LEUKOCYTE_MIGRATION
LIN_NEG_VS_BCELL_UP
NEGATIVE_REGULATION_OF_TRANSMEMBRANE_TRANSPORT
RAG2_KO_VS_RAG2_AND_ETS1_KO_NK_CELL_UP
REGULATION_OF_FATTY_ACID_METABOLIC_PROCESS
ROSIGLITAZONE_IL4_VS_IL4_STIM_MACROPHAGE
Rosacea
Senile_cataract
Asthma_with_exacerbation
Electrolyte_imbalance
Abdominal_pain
Angina_pectoris
LOF_Filter_2
DRUG_METABOLIC_PROCESS
DRUG_METABOLIC_PROCESS
DRUG_METABOLISM_CYTOCHROME_P450
DRUG_METABOLISM_CYTOCHROME_P450
DRUG_METABOLISM_CYTOCHROME_P450
DRUG_METABOLISM_OTHER_ENZYMES
GLUTAMINE_FAMILY_AMINO_ACID_METABOLIC_PROCESS
METABOLISM_OF_XENOBIOTICS_BY_CYTOCHROME_P450
RETINOL_METABOLISM
VMYOD_Q6_01
Other_peripheral_nerve_disorders
Psoriatic_arthropathy
Cervical_radiculitis
Psoriatic_arthropathy
Renal_failure_NOS
Pain_in_joint
Pain_in_joint
Psoriatic_arthropathy
Psoriatic_arthropathy
Acne
LOF_Filter_3
ACTION_POTENTIAL
COENZYME_METABOLIC_PROCESS
CTRL_VS_TCELL_MEMBRANES_ACT_MAST_CELL_PRETREAT_A3R_INH_UP
GP33_VS_GP276_LCMV_SPECIFIC_EXHAUSTED_CD8_TCELL_UP
RETINOL_METABOLISM
TH0_VS_TGFB_IL6_TH17_ACT_CD4_TCELL_30H_UP
Macular_degeneration,_wet
Sprains_and_strains_of_back_and_neck
Sicca_syndrome
Chronic_airway_obstruction
Chronic_ulcer_of_leg_or_foot
Disturbance_of_skin_sensation
NON_LOF_Variants
AMINO_ACID_TRANSPORT
CTRL_VS_ANTI_VBETA5_DP_THYMOCYTE_DN
DICARBOXYLIC_ACID_TRANSPORT
FOCAL_ADHESION
ISOPRENOID_METABOLIC_PROCESS
POSITIVE_REGULATION_OF_DNA_METABOLIC_PROCESS
REGULATION_OF_DNA_METABOLIC_PROCESS
TREG_PLN_VS_CD24HI_TREG_THYMUS_UP
VICSBP_Q6
Type_1_diabetes
Umbilical_hernia
Polycystic_ovaries
Diabetic_retinopathy
Back_pain
Sleep_apnea
Sleep_apnea
Nonrheumatic_mitral_valve_disorders
Herpes_simplex

Supplementary Figure 9: The top 3 enriched gene sets along with the genes that are found to be enriched in these pathways. The size of the points corresponds to the normalized enrichment score, and the color corresponds to the Filter type from the tests. X-axis labels are gene names and y-axis labels are clinical lab measures or ICD-9 phenotype categories)

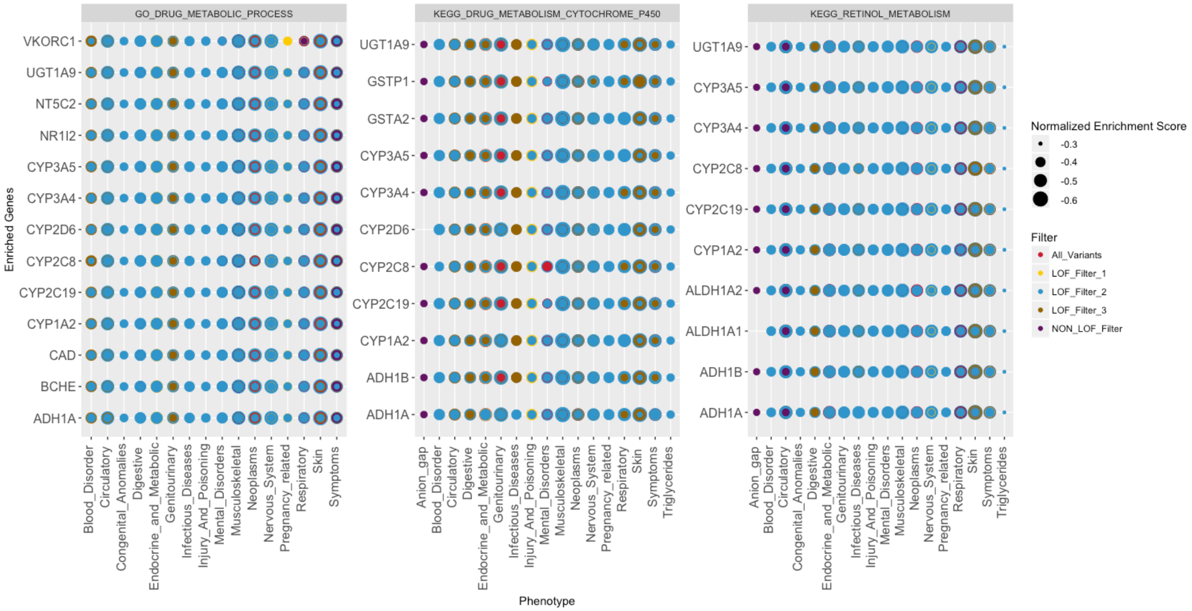

Supplementary Table 1: Bonferroni Significant Results

Phenotype	Category	Gene	Num Cases	Num Controls	Num Vars	Case Vars	Control Vars	Case Capacity	Control Capacity	Analysis source	Beta	Pval	SE	Novel or Known
Platelet Counts	Platelet Counts	*TUBB1*	0	35786	844	0	844	0	5648666	Filter 2	-34.1	7.85E-11	5.24	Known
Alanine AminoTransferase	Alanine AminoTransferase	*GPT*	0	34350	910	0	910	0	8808146	Filter 3	-0.72	3.29E-83	0.03	Known
Alanine AminoTransferase	Alanine AminoTransferase	*GPT*	0	34350	348	0	348	0	5343252	Filter 2	-0.84	3.29E-83	0.04	Known
Alanine AminoTransferase	Alanine AminoTransferase	*GPT*	0	34350	1697	0	1697	0	15643938	AllVar	-0.43	2.80E-78	0.02	Known
Bilirubin	Bilirubin	*UGT1A1*	0	33769	502	0	502	0	7833072	Filter 1	0.29	4.61E-17	0.03	Known
Bilirubin	Bilirubin	*UGT1A1*	0	33769	378	0	378	0	7022704	Filter 3	0.3	1.42E-16	0.04	Known
HDL	HDL	*ABCA1*	0	29665	587	0	587	0	7528332	Filter 2	-0.18	3.81E-15	0.02	Known
Bilirubin	Bilirubin	*UGT1A3*	0	33769	860	0	860	0	9048832	Filter 3	0.24	5.85E-15	0.03	Known
Bilirubin	Bilirubin	*UGT1A3*	0	33769	1142	0	1142	0	11547646	Filter 1	0.2	9.54E-14	0.03	Known
Bilirubin	Bilirubin	*UGT1A5*	0	33769	1843	0	1843	0	13100988	Filter 3	0.19	1.99E-13	0.03	Known
Bilirubin	Bilirubin	*UGT1A4*	0	33769	1313	0	1313	0	11345066	Filter 3	0.19	2.02E-12	0.03	Known
Bilirubin	Bilirubin	*UGT1A6*	0	33769	1972	0	1972	0	15734846	Filter 3	0.16	5.71E-12	0.02	Known

Bilirubin	Bilirubin	*UGT1A7*	0	33769	2284	0	2284	0	17558210	Filter 3	0.15	2.00E-11	0.02	Known
Bilirubin	Bilirubin	*UGT1A5*	0	33769	2663	0	2663	0	18233706	Filter 1	0.13	1.37E-10	0.02	Known
Bilirubin	Bilirubin	*UGT1A6*	0	33769	2871	0	2871	0	21745420	Filter 1	0.12	1.60E-10	0.02	Known
Bilirubin	Bilirubin	*UGT1A10*	0	33769	2539	0	2539	0	19584322	Filter 3	0.13	8.15E-10	0.02	Known
Bilirubin	Bilirubin	*UGT1A*	0	33769	2539	0	2539	0	19584322	Filter 3	0.13	8.15E-10	0.02	Known
Bilirubin	Bilirubin	*UGT1A8*	0	33769	2539	0	2539	0	19584322	Filter 3	0.13	8.15E-10	0.02	Known
Bilirubin	Bilirubin	*UGT1A9*	0	33769	2539	0	2539	0	19584322	Filter 3	0.13	8.15E-10	0.02	Known
HDL	HDL	*ABCA1*	0	29665	1995	0	1995	0	19327520	Filter 3	-0.08	9.59E-10	0.01	Known
Hypercalcemia	Endocrine and Metabolic	*CASR*	252	37742	94	11	83	22136	3313030	Filter 2	3.89	1.34E-22	0.40	Known
Alcohol abuse, unspecified	Mental Disorders	*ADRA2B*	149	37775	78	4	74	2948	748612	Filter 2	4.59	3.88E-10	0.73	Known
Other specified erythematous conditions	Skin	*RBP1*	184	37318	207	7	200	14286	2898302	Filter 1	3.26	1.28E-09	0.54	Known
Other specified erythematous conditions	Skin	*RBP1*	184	37318	235	7	228	17176	3484988	AllVar	3.05	9.82E-09	0.53	Known
Hypercalcemia	Endocrine and Metabolic	*CASR*	252	37742	337	12	325	52322	7833962	Filter 3	2.34	1.32E-08	0.41	Known
Hypercalcemia	Endocrine and Metabolic	*CASR*	252	37742	633	17	616	61392	9192314	Filter 1	2.19	1.74E-08	0.39	Known

Tachycardia, unspecified	Symptoms	*KCNJ12*	226	36588	429	6	423	12158	1965614	Filter 2	3.37	2.98E-08	0.61 Known
Vitreous hemorrhage	Nervous System	*PF4*	210	38174	46	3	43	2520	458052	VarOnly	5.53	3.54E-08	1.00 Known
Lesion of ulnar nerve	Nervous System	*CACNB1*	188	37821	909	9	900	31878	6413544	Filter 1	2.63	5.61E-08	0.49 Known
Chronic pain syndrome	Nervous System	*SERPINA5*	217	37906	111	4	107	9970	1740536	Filter 2	3.51	5.80E-08	0.65 Known
Unspecified disorder of kidney and ureter	Genitourinary	*AKR1C3*	339	36914	343	10	333	18960	2064600	Filter 2	3.17	7.56E-08	0.59 Known
Postinflammatory pulmonary fibrosis	Respiratory	*AGTR1*	193	38077	531	8	523	20138	3943358	Filter 3	2.94	1.03E-07	0.55 Known
Ptosis of eyelid, unspecified	Nervous System	*S100B*	253	37877	39	3	36	9108	1363556	Filter 1	3.66	1.04E-07	0.69 Known
Attention deficit disorder with hyperactivity	Mental Disorders	*VEGFA*	178	38209	69	6	63	9098	1959638	Filter 2	2.7	1.08E-07	0.51 Known
WBC Counts	WBC Counts	*GLCCI1*	0	36587	82	0	82	0	2122688	Filter 2	-0.33	2.33E-13	0.04 Novel
Unspecified sinusitis (chronic)	Respiratory	*TACR1*	578	35475	267	15	252	77318	4746300	Filter 3	2.35	2.02E-10	0.37 Novel
Orthostatic hypotension	Circulatory	*GUCY1A2*	186	37300	437	9	428	29498	5921056	Filter 3	2.81	7.79E-10	0.46 Novel
Unspecified sinusitis (chronic)	Respiratory	*TACR1*	578	35475	275	15	260	83098	5100974	Filter 1	2.26	7.98E-10	0.37 Novel

Thoracic aneurysm without mention of rupture	Circulatory	*GSK3B*	223	38131	228	7	221	11596	1981910	VarOnly	3.89	1.08E-09	0.64	Novel
Adjustment disorder with anxiety	Mental Disorders	*NPR3*	206	37764	718	10	708	39226	7194718	Filter 3	2.45	1.21E-09	0.40	Novel
Other closed fractures of distal end of radius (alone)	Injury And Poisoning	*THBD*	183	38128	1269	13	1256	57000	11972710	AllVar	2.11	3.54E-09	0.36	Novel
Psoriatic arthropathy	Skin	*SSTR2*	172	38308	121	5	116	18904	4210310	Filter 3	2.91	3.78E-09	0.49	Novel
Proliferative diabetic retinopathy	Nervous System	*GSTA2*	243	38194	571	7	564	14580	2291582	Filter 1	3.54	3.81E-09	0.60	Novel
Chronic lymphocytic thyroiditis	Endocrine and Metabolic	*BGLAP*	222	38080	23	2	21	3988	681930	Filter 2	5.22	5.02E-09	0.89	Novel
Sacroiliitis, not elsewhere classified	Musculoskel etal	*KCNJ14*	708	36820	1763	45	1718	2E+05	9621778	AllVar	1.63	5.13E-09	0.28	Novel
Malignant neoplasm of upper lobe, bronchus or lung	Neoplasms	*REN*	144	38318	214	4	210	8912	2373662	VarOnly	3.63	5.17E-09	0.62	Novel

Abnormal glucose tolerance of mother, antepartum condition or complication	Pregnancy related	*PTGR2*	207	38106	141	5	136	11158	2054380	Filter 2	4.02	5.49E-09	0.69	Novel
Deviated nasal septum	Respiratory	*FKBP1A*	227	37572	22	3	19	4432	738012	AllVar	4.27	6.79E-09	0.74	Novel
Orthostatic hypotension	Circulatory	*GUCY1A2*	186	37300	553	10	543	36792	7386224	Filter 1	2.54	7.11E-09	0.44	Novel
Postlaminecto my syndrome, lumbar region	Musculoskel etal	*SERPINB2*	578	37622	755	23	732	97076	6317542	Filter 3	1.97	8.02E-09	0.34	Novel
Hyposmolality and/or hyponatremia	Endocrine and Metabolic	*COX6C*	239	36793	10	3	7	2384	367652	Filter 2	6.37	8.28E-09	1.10	Novel
Nonspecific abnormal results of function study of liver	Symptoms	*BDKRB2*	591	36386	253	17	236	46478	2856568	VarOnly	2.44	8.90E-09	0.42	Novel
Urgency of urination	Symptoms	*HTR1D*	178	37035	420	10	410	29138	6060614	Filter 1	2.73	9.65E-09	0.48	Novel
Panniculitis, unspecified site	Musculoskel etal	*ARVCF*	308	38015	238	8	230	28788	3552416	AllVar	2.92	1.02E-08	0.51	Novel
Psoriatic arthropathy	Skin	*SSTR2*	172	38308	136	5	131	21648	4820996	Filter 1	2.77	1.12E-08	0.49	Novel

Temporomandibular joint disorders, unspecified	Digestive	*PTGDR2*	218	37188	260	8	252	24316	4159712	Filter 3	2.84	1.36E-08	0.50	Novel
Other tenosynovitis of hand and wrist	Musculoskeletal	*CACNA1H*	164	36967	7377	47	7330	3E+05	58268070	Filter 1	1.35	1.50E-08	0.24	Novel
Sinoatrial node dysfunction	Circulatory	*LIPF*	268	38020	150	3	147	8558	1214432	Filter 2	3.96	1.56E-08	0.70	Novel
Other syndromes affecting cervical region	Musculoskeletal	*HTR3B*	149	38163	1152	12	1140	29198	7477184	Filter 1	2.27	1.77E-08	0.40	Novel
Lesion of ulnar nerve	Nervous System	*CACNB1*	188	37821	718	9	709	30000	6035500	Filter 3	2.71	1.79E-08	0.48	Novel
Ostium secundum type atrial septal defect	Congenital Anomalies	*HSD11B1*	164	38148	80	3	77	7216	1678092	Filter 3	3.69	1.89E-08	0.66	Novel
Proliferative diabetic retinopathy	Nervous System	*GSTA2*	243	38194	550	6	544	11178	1756900	Filter 3	3.7	1.92E-08	0.66	Novel
Sinoatrial node dysfunction	Circulatory	*CHRNB4*	268	38020	637	16	621	20020	2824836	VarOnly	3.3	2.26E-08	0.59	Novel
Postinflammatory pulmonary fibrosis	Respiratory	*AGTR1*	193	38077	344	6	338	9922	1939538	Filter 2	3.45	2.28E-08	0.62	Novel

Degeneration of intervertebral disc, site unspecified	Musculoskeletal	*SCN7A*	178	38006	612	11	601	30914	6594694	Filter 2	2.56	2.45E-08	0.46	Novel
Bipolar disorder, unspecified	Mental Disorders	*PDXK*	427	37727	226	7	219	31392	2772698	VarOnly	2.82	2.47E-08	0.51	Novel
Varicose veins of lower extremities with ulcer	Circulatory	*TYMS*	159	38153	741	11	730	19148	4603708	Filter 1	2.79	2.49E-08	0.50	Novel
Chronic diastolic heart failure	Circulatory	*KCNJ14*	270	37673	884	16	868	18518	2592428	VarOnly	3.22	2.62E-08	0.58	Novel
Other tenosynovitis of hand and wrist	Musculoskeletal	*CACNA1H*	164	36967	12883	76	12807	4E+05	91080326	AllVar	1.15	3.69E-08	0.21	Novel
Endothelial corneal dystrophy	Nervous System	*NFKB1*	155	38251	323	7	316	30334	7491708	Filter 3	2.71	3.73E-08	0.49	Novel
Closed fracture of metatarsal bone(s)	Injury And Poisoning	*NPR2*	147	38119	322	8	314	18736	4858588	VarOnly	2.99	4.24E-08	0.55	Novel
Bunion	Musculoskeletal	*DBH-AS1*	155	37559	301	4	297	3948	954562	Filter 2	4.52	4.46E-08	0.83	Novel

Cerebral artery occlusion, unspecified with cerebral infarction	Circulatory	*GNRHR*	325	37467	511	11	500	8878	1022112	Filter 2	3.76	4.85E-08	0.69	Novel
Chronic obstructive asthma, unspecified	Respiratory	*PTGS2*	183	37841	164	5	159	15720	3253268	VarOnly	3.1	4.91E-08	0.57	Novel
Alcohol abuse, unspecified	Mental Disorders	*DNMT1*	149	37775	113	4	109	12206	3092700	Filter 2	3.1	5.88E-08	0.57	Novel
Unspecified pleural effusion	Respiratory	*GHR*	294	37163	932	17	915	52316	6613700	Filter 3	2.23	6.01E-08	0.41	Novel
Acute maxillary sinusitis	Respiratory	*TFPI*	159	37059	11	2	9	2844	662252	Filter 2	4.4	6.44E-08	0.81	Novel
Sensorineural hearing loss, unspecified	Nervous System	*FOS*	899	35965	467	21	446	36530	1464190	Filter 2	3.03	6.73E-08	0.56	Novel
Degeneration of intervertebral disc, site unspecified	Musculoskeletal	*KCNJ11*	178	38006	470	8	462	21966	4699252	VarOnly	2.68	7.09E-08	0.50	Novel
Dehydration	Endocrine and Metabolic	*SLC22A6*	162	36308	650	7	643	39032	8742788	Filter 1	2.53	7.24E-08	0.47	Novel
Deviated nasal septum	Respiratory	*ANPEP*	227	37572	365	8	357	26864	4445828	Filter 2	2.77	7.79E-08	0.52	Novel

Solitary pulmonary nodule	Symptoms	*CACNA2D3-AS1*	201	37675	31	3	28	5224	979330	VarOnly	3.91	7.93E-08	0.73	Novel
Ocular hypertension	Nervous System	*ADH1C*	446	37452	1453	25	1428	46384	3894932	VarOnly	2.49	7.98E-08	0.46	Novel
Cellulitis and abscess of foot, except toes	Skin	*ACSL3*	209	37324	1192	19	1173	20026	3576910	VarOnly	2.99	8.23E-08	0.56	Novel
Osteoarthrosis, generalized, site unspecified	Musculoskeletal	*RARA*	2813	32960	350	47	303	5E+05	5508298	Filter 3	1.5	8.69E-08	0.28	Novel
Unspecified episodic mood disorder	Mental Disorders	*SELP*	235	37548	239	6	233	25844	4129570	Filter 2	2.78	9.80E-08	0.52	Novel
Difficulty in walking	Musculoskeletal	*CA3*	196	37992	62	4	58	10174	1972856	Filter 2	3.54	9.98E-08	0.67	Novel
Atherosclerosis of native arteries of the extremities, unspecified	Circulatory	*GABBR2*	305	37502	38	4	34	13022	1608186	Filter 2	3.31	1.03E-07	0.62	Novel
Pain in thoracic spine	Musculoskeletal	*NPPB*	148	37332	701	9	692	2068	522400	VarOnly	5.13	1.04E-07	0.96	Novel

Migraine, unspecified, without mention of intractable migraine without mention of status migrainosus	Nervous System	*NR1I2*	1395	35151	961	56	905	1E+05	2948762	VarOnly	2.09	1.04E-07	0.39	Novel
Adjustment disorder with anxiety	Mental Disorders	*NPR3*	206	37764	820	10	810	52404	9610368	Filter 1	2.15	1.08E-07	0.41	Novel

Supplementary Table 2: Genes and their positions

Gene	Chr	startpos	endpos
A2M	12	9220304	9268558
ABCA1	9	107543283	107690527
ABCB1	7	87133179	87342639
ABCB11	2	169779312	169891400
ABCC1	16	16043434	16236931
ABCC2	10	101542355	101612333
ABCC8	11	17414432	17498392
ABCC9	12	21950323	22094797
ACE	17	61554422	61575741
ACHE	7	100487615	100494306
ACSL3	2	223725702	223808119
ACY1	3	52017300	52023218
ACY3	11	67410026	67418278
ADA	20	43248163	43280874
ADH1A	4	100197523	100212185
ADH1B	4	100227544	100242599
ADH1C	4	100257649	100274202
ADK	10	75910943	76469061
ADORA1	1	203096833	203136533
ADORA2A	22	24819565	24838328
ADORA2B	17	15831097	15879210
ADORA3	1	112042051	112046743
ADRA1A	8	26600364	26728525
ADRA1B	5	159343740	159413185
ADRA1D	20	4201278	4229659
ADRA2A	10	112836790	112840665
ADRA2B	2	96778623	96781984
ADRA2C	4	3768296	3770253
ADRB1	10	115803806	115806667
ADRB2	5	148206156	148208197
ADRB3	8	37820513	37824184
ADSS	1	244571794	244615436
ADSSL1	14	105190534	105213662
AGTR1	3	148415658	148460790
AGXT	2	241808162	241818536
AHR	7	17338276	17385775
AKR1A1	1	46016455	46035723
AKR1B1	7	134127103	134143888
AKR1B10	7	134212344	134226166
AKR1C1	10	5005454	5020158

AKR1C2	10	5029967	5060225
AKR1C3	10	5090958	5149878
AKR1D1	7	137761178	137803050
ALB	4	74269972	74287129
ALDH18A1	10	97365686	97416567
ALDH1A1	9	75515578	75568233
ALDH1A2	15	58245622	58358121
ALKBH2	12	109525993	109531436
ALKBH3	11	43902357	43941825
ALOX5	10	45869624	45941569
AMN	14	103388885	103397184
AMY2A	1	104159954	104168402
ANG	14	21152336	21162345
ANPEP	15	90328126	90358086
ANXA1	9	75766647	75785309
ANXA2	15	60639350	60690185
AOC3	17	41003140	41010147
APRT	16	88875877	88878342
AQP1	7	30951415	30965131
ARF1	1	228270361	228286913
ARG1	6	131894343	131905472
ASGR2	17	7004641	7018841
ASL	7	65540776	65558330
ASNS	7	97481429	97501854
ASPA	17	3377404	3402700
ASPH	8	62413115	62627199
ASRGL1	11	62104774	62168850
ASS1	9	133320094	133376661
ATF1	12	51157493	51214950
ATF2	2	175936978	176032934
ATF3	1	212738676	212794119
ATF4	22	39916564	39918691
ATF5	19	50431959	50437193
ATF6	1	161736034	161933860
ATF7	12	53901640	54020199
ATM	11	108093227	108239829
ATP1A1	1	116914807	116947396
ATP2C1	3	130569327	130735556
ATP4A	19	36041095	36054560
ATP6V1A	3	113465866	113530905
AVPR1A	12	63536067	63547640
AVPR1B	1	206224283	206231482

BAAT	9	104122699	104147287
BBOX1	11	27062252	27149354
BCHE	3	165490692	165555260
BCL2	18	60790579	60987011
BDKRB2	14	96671135	96710666
BGLAP	1	156211951	156213123
BHMT	5	78407604	78428113
C1QA	1	22963118	22966175
C1QB	1	22979672	22988045
C1QC	1	22970118	22974603
C1R	12	7187513	7189412
C1S	12	7167980	7178336
C3	19	6677846	6720693
C4A	6	31949834	31970457
C4B	6	31982572	32003195
C5	9	123714613	123837203
C8G	9	139839327	139841426
CA1	8	86240458	86290383
CA10	17	49707674	50237377
CA11	19	49141272	49149451
CA12	15	63615730	63674309
CA13	8	86157716	86196302
CA14	1	150229554	150237478
CA2	8	86376131	86393721
CA3	8	86351056	86361269
CA4	17	58227302	58236907
CA6	1	9005844	9035151
CA7	16	66878282	66888052
CA8	8	61099906	61193954
CA9	9	35673915	35681156
CACNA1A	19	13317256	13617274
CACNA1B	9	140772241	141019076
CACNA1C	12	2079952	2807115
CACNA1D	3	53529003	53847178
CACNA1G	17	48638429	48706986
CACNA1H	16	1203241	1271772
CACNA1I	22	39953974	40085744
CACNA1S	1	201008635	201081694
CACNA2D1	7	81575760	82073122
CACNA2D2	3	50400044	50541675
CACNA2D3	3	54156620	55108584
CACNB1	17	37329709	37353956

CACNB2	10	18429606	18830688
CACNB3	12	49208215	49222726
CACNB4	2	152689285	152956502
CACNG1	17	65040652	65052913
CAD	2	27440258	27466660
CALCR	7	93053799	93204042
CALM1	14	90863327	90874619
CALR	19	13049414	13055304
CALY	10	135137576	135150475
CANX	5	179105647	179158642
CARTPT	5	71014990	71016875
CASR	3	121902514	122005350
CAST	5	95997741	96110387
CBS	21	44473301	44496978
CCL2	17	32582296	32584222
CCR5	3	46411633	46417697
CD44	11	35160417	35253949
CD55	1	207494817	207534311
CDO1	5	115140430	115152651
CDO1	5	115149317	115151808
CES1	16	55836763	55867098
CFTR	7	117118394	117308719
CHAT	10	50817141	50873150
CHRFAM7A	15	30649973	30686052
CHRM1	11	62676151	62689333
CHRM2	7	136553399	136705002
CHRM3	1	239549818	239550527
CHRM3	1	239549865	240074762
CHRM4	11	46406342	46408158
CHRM5	15	34260446	34357295
CHRNA1	2	175612320	175629200
CHRNA10	11	3686817	3694438
CHRNA2	8	27317278	27336813
CHRNA3	15	78885394	78913637
CHRNA4	20	61974662	61992748
CHRNA5	15	78857862	78887611
CHRNA6	8	42607763	42623929
CHRNA7	15	32322686	32464722
CHRNA9	4	40337346	40357234
CHRNB1	17	7348406	7360932
CHRNB2	1	154540257	154552354
CHRNB3	8	42552562	42592550

CHRNB4	15	78915639	78947928
CHRND	2	233390870	233401375
CHRNE	17	4801064	4806423
CHRNG	2	233404437	233411038
CHUK	10	101945870	101989367
CKB	14	103985995	103989196
CKM	19	45809671	45826235
CLCN2	3	184063973	184079439
CLCNKA	1	16348486	16360545
CLEC3B	3	45067759	45077563
CNR1	6	88849583	88876464
CNR2	1	24199953	24239817
COL1A1	17	48261457	48279009
COL1A2	7	94023873	94060544
COL2A1	12	48366748	48419069
COL3A1	2	189839099	189877472
COMP	19	18893583	18902114
COMT	22	19929263	19957498
COX6B1	19	36139125	36149686
COX6C	8	100890223	100906242
CP	3	148880197	148939837
CPB1	3	148508967	148577979
CPE	4	166300097	166419482
CRH	8	67088612	67090846
CRYZ	1	75171166	75199092
CSAD	12	53551447	53574693
CTDSP1	2	219263061	219270664
CTH	1	70876901	70905797
CTRB1	16	75252884	75258822
CTSD	11	1773982	1785222
CUBN	10	16865965	17172248
CXCL12	10	44865601	44880545
CXCR1	2	219027568	219031716
CXCR4	2	136871919	136875725
CYBA	16	88709697	88717493
CYP19A1	15	51500254	51630795
CYP1A2	15	75041184	75048941
CYP21A2	6	32006093	32009447
CYP2C19	10	96522438	96612962
CYP2C8	10	96796529	96829255
CYP2D6	22	42521661	42531387
CYP3A4	7	99354583	99381811

CYP3A5	7	99245812	99277649
CYP51A1	7	91741463	91764059
CYSLTR2	13	49280951	49283498
DBH	9	136501485	136524466
DDC	7	50526134	50633154
DHFR	5	79922045	79950800
DLG4	17	7093209	7123369
DNMT1	19	10244021	10305755
DNPEP	2	220238180	220264739
DPP4	2	162848751	162931052
DRD1	5	174867675	174871163
DRD2	11	113280317	113346001
DRD3	3	113846644	113918254
DRD4	11	637305	640706
DRD5	4	9783258	9785633
EDNRA	4	148402069	148466106
EDNRB	13	78469616	78549664
EGF	4	110834039	110934118
EGFR	7	55086725	55275031
EGLN1	1	231499497	231560790
EGLN2	19	41305048	41314346
EGLN3	14	34393421	34420289
ELN	7	73442102	73484237
ENPEP	4	111397229	111484493
ENPP1	6	132129156	132216295
EPOR	19	11487881	11495018
EPRS	1	220141942	220220000
EPX	17	56270089	56282535
ESR1	6	152011631	152424409
ESR2	14	64639643	64805268
ESRRA	11	64073000	64084215
ESRRG	1	216676588	217311097
F10	13	113777113	113803843
F11	4	187187099	187210835
F12	5	176829139	176843525
F2	11	46740742	46761056
F2R	5	76011868	76031595
F5	1	169481192	169555769
F7	13	113760102	113774995
FAAH	1	46859939	46879520
FABP6	5	159614374	159665729
FADS1	11	61567097	61584529

FASN	17	80036214	80056165
FCER1A	1	159253678	159278014
FCGR1A	1	149752831	149764074
FCGR2A	1	161475205	161493838
FCGR2B	1	161632905	161648444
FCGR2C	1	161551129	161571010
FCGR3A	1	161511549	161520413
FCGR3B	1	161592986	161601753
FDPS	1	155278539	155290457
FFAR1	19	35842445	35843367
FGA	4	155504280	155511897
FGB	4	155484132	155493915
FGF1	5	141971743	142077635
FGF2	4	123747863	123819390
FGF4	11	69573811	69590171
FGG	4	155525286	155533902
FKBP1A	20	1349621	1373816
FOLH1	11	49168187	49230222
FOS	14	75745481	75748937
FPR1	19	52249023	52255150
FSHR	2	49093574	49381666
FTCD	21	47556065	47575770
FXYD2	11	117690790	117698807
GAA	17	78075330	78093680
GABBR1	6	29570005	29600962
GABBR2	9	101050364	101471479
GABRA1	5	161274197	161326965
GABRA2	4	46245565	46392056
GABRA4	4	46920917	46996424
GABRA5	15	27111866	27194357
GABRA6	5	161112658	161129598
GABRB1	4	46995875	47429360
GABRB2	5	160715426	160975130
GABRB3	15	26788693	27018935
GABRD	1	1950768	1962192
GABRG1	4	46037786	46126082
GABRG2	5	161494648	161582545
GABRG3	15	27216429	27779041
GABRP	5	170199570	170241051
GABRR1	6	89887223	89941007
GABRR2	6	89965780	90025018
GABRR3	3	97705527	97754148

GAD1	2	171669720	171717661
GAD2	10	26505236	26593491
GAMT	19	1397025	1401569
GANAB	11	62392298	62414198
GANC	15	42565856	42645864
GARS	7	30634181	30673649
GCGR	17	79762008	79771889
GCLC	6	53362139	53409927
GCLM	1	94350761	94375154
GGCX	2	85771978	85788657
GGPS1	1	235491753	235507847
GHR	5	42423877	42721980
GHRHR	7	31003636	31019146
GJA1	6	121756723	121770890
GLO1	6	38643701	38670952
GLP1R	6	39016557	39059079
GLP2R	17	9728860	9795421
GLRA1	5	151202074	151304427
GLRA3	4	175545292	175750466
GLRB	4	157997277	158093251
GLUL	1	182347228	182361341
GLYAT	11	58476230	58499447
GLYATL1	11	58695102	58724547
GLYATL2	11	58601538	58612281
GMPS	3	155587219	155657397
GNG2	14	52313849	52436518
GNMT	6	42907153	42931618
GNRHR	4	68603093	68621804
GNRHR2	1	145509752	145516076
GP9	3	128774292	128781254
GPR18	13	99906967	99914702
GPRC5A	12	13043956	13066600
GPT	8	145727604	145732557
GPT2	16	46918308	46965201
GRIA1	5	152869256	153193433
GRIA2	4	158141736	158287227
GRIA4	11	105480721	105852819
GRM1	6	146285905	146758734
GRM4	6	33986420	34123399
GRM5	11	88237744	88799129
GRM7	3	6902784	7783218
GRM8	7	126078652	126893348

GSK3B	3	119540168	119813264
GSS	20	33516236	33543795
GSTA2	6	52614885	52628361
GSTP1	11	67351066	67354124
GUCY1A2	11	106544738	106889171
GUCY2C	12	14765566	14849552
HABP2	10	115310590	115349361
HABP4	9	99212437	99253618
HAPLN1	5	82934017	83016896
HAPLN3	15	89420519	89438857
HAPLN4	19	19366450	19373596
HBA1	16	226650	227521
HDAC2	6	114257320	114292359
HDAC9	7	18126565	19039144
HGS	17	79650962	79669151
HIF1A	14	62162119	62214977
HMGCR	5	74632159	74657926
HMMR	5	162887517	162918951
HNMT	2	138721808	138773934
HPGDS	4	95219707	95264027
HRH1	3	11178779	11304939
HRH2	5	175084799	175137759
HRH3	20	60789822	60795334
HRH4	18	22040532	22067391
HS3ST3A1	17	13397349	13505291
HSD11B1	1	209859525	209908295
HSP90AA1	14	102547075	102606096
HSP90B1	12	104324112	104341708
HSPA5	9	127997127	128003666
HTR1A	5	63255875	63258119
HTR1B	6	78171948	78173120
HTR1D	1	23518388	23521222
HTR1E	6	87647024	87726397
HTR1F	3	87841917	88044006
HTR2A	13	47405677	47471211
HTR2B	2	231972944	231990492
HTR3A	11	113845797	113861035
HTR3B	11	113769645	113817283
HTR3C	3	183770835	183778461
HTR3D	3	183749332	183757157
HTR3E	3	183814852	183824783
HTR4	5	147830595	148034090

HTR5A	7	154862034	154879102
HTR6	1	19991780	20007459
HTR7	10	92500575	92617671
ICAM1	19	10381517	10397291
IDE	10	94211441	94333852
IFNG	12	68548550	68553521
IGF1R	15	99191768	99507759
IGF2R	6	160390131	160527583
IGFBP3	7	45951844	45960871
IGFBP7	4	57897237	57976551
IKBKB	8	42128820	42190171
IL2	4	123372625	123377650
IL4I1	19	50392911	50432796
IL5	5	131877136	131892582
IMPA1	8	82569151	82598589
IMPA2	18	11981427	12030885
IMPDH1	7	128032331	128050041
IMPG2	3	100941390	101039419
INSR	19	7112266	7294011
ISYNA1	19	18545198	18549111
ITGA2B	17	42449549	42466873
ITGA4	2	182321619	182402474
ITGAL	16	30483983	30534506
ITGB2	21	46305864	46348788
ITGB3	17	45331208	45390077
ITPA	20	3189514	3208095
JDP2	14	75894509	75939404
JUN	1	59246463	59249785
KARS	16	75661622	75681585
KCNA1	12	5019073	5027422
KCNA10	1	111059839	111061797
KCNA2	1	111136202	111174136
KCNA3	1	111196182	111217655
KCNA4	11	30031288	30038577
KCNA5	12	5153085	5155954
KCNA6	12	4916051	4960280
KCNA7	19	49570675	49576198
KCNB1	20	47980414	48100579
KCNB2	8	73449626	73850584
KCNC1	11	17757457	17885835
KCNC2	12	75433858	75603528
KCNC3	19	50815199	50836772

KCND2	7	119913695	120390387
KCND3	1	112318444	112532198
KCNE1	21	35791013	35884573
KCNH2	7	150642044	150675402
KCNH6	17	61600695	61626338
KCNH7	2	163227917	163695257
KCNJ1	11	128707909	128737268
KCNJ10	1	160007257	160040051
KCNJ11	11	17386371	17410878
KCNJ12	17	21279699	21323184
KCNJ14	19	48958964	48969367
KCNJ15	21	39601837	39675048
KCNJ3	2	155555093	155714864
KCNJ5	11	128761313	128791058
KCNJ6	21	38989784	39292034
KCNJ8	12	21917889	21928436
KCNK1	1	233749750	233808258
KCNK2	1	215175982	215410436
KCNK3	2	26915581	26954066
KCNK6	19	38810454	38819654
KCNK9	8	140613081	140715311
KCNMA1	10	78629359	79397577
KCNMB1	5	169805165	169816681
KCNMB2	3	178254086	178562217
KCNMB3	3	178957537	178984838
KCNMB4	12	70760003	70828072
KCNN1	19	18062111	18109930
KCNN2	5	113391703	113832197
KCNN3	1	154669938	154842754
KCNN4	19	44270685	44286291
KCNQ1	11	2466221	2870340
KCNQ2	20	62031561	62103993
KCNQ3	8	133133105	133493004
KCNQ4	1	41249684	41306124
KCNQ5	6	73331008	73908574
KLK1	19	51322402	51327043
LAYN	11	111409710	111432470
LCT	2	136545415	136594750
LGSN	6	63985856	64277826
LHCGR	2	48913913	48982880
LIPF	10	90424146	90438572
LMAN1	18	56995055	57026508

LPA	6	160952515	161087407
LPL	8	19796582	19824770
LRP1	12	57522282	57607142
LRP2	2	169983619	170219123
LSM6	4	147096835	147111213
LTA4H	12	96394531	96437298
LTF	3	46477496	46506632
LTF	3	46509625	46526724
LYZ	12	69742134	69748013
MAPK1	22	22113946	22221970
MAPK3	16	30125426	30134630
MC2R	18	13882043	13915706
MCFD2	2	47129009	47168994
METAP2	12	95867822	95909615
MGMT	10	131265454	131566306
MIF	22	24236565	24237409
MLNR	13	49794474	49796513
MMACHC	1	45965856	45976739
MMP12	11	102733464	102745764
MMP2	16	55512742	55540603
MPL	1	43803089	43820135
MPO	17	56347216	56361674
MS4A2	11	59855734	59865940
MTHFR	1	11845787	11866160
MTNR1A	4	187454154	187476537
MTNR1B	11	92702789	92719406
MTOR	1	11166588	11322614
MTR	1	236957604	237067281
MTRR	5	7851299	7901237
MTTP	4	100485235	100545154
MYC	8	128748315	128753680
NADSYN1	11	71164217	71212584
NAGA	22	42454338	42466875
NAGK	2	71295371	71305998
NAGLU	17	40687951	40696467
NAGPA	16	5074845	5083942
NCAN	19	19322773	19363061
NCF1	7	74188309	74203720
NCF2	1	183524697	183560056
NCF4	22	37257030	37274059
NDUFC2	11	77779393	77791265
NEU1	6	31826829	31830709

NEU2	2	233887766	233900242
NFKB1	4	103422486	103538459
NGF	1	115828537	115881005
NID1	1	236139130	236228481
NISCH	3	52489524	52527088
NOV	8	120428552	120436678
NPC1L1	7	44552134	44580929
NPPB	1	11917521	11918992
NPR1	1	153651093	153666468
NPR2	9	35752942	35809728
NPR3	5	32689182	32791830
NPSR1	7	34697851	34917944
NQO1	16	69743304	69760571
NQO2	6	2988874	3020110
NR1H4	12	100867537	100957645
NR1I2	3	119499331	119537332
NR3C1	5	142657496	142815077
NR3C2	4	148999915	149366430
NRP1	10	33466419	33623833
NT5C2	10	104847774	104953063
NT5E	6	86159302	86205509
NTRK1	1	156785542	156851642
NTRK2	9	87283373	87641985
NTSR2	2	11798304	11810329
OAZ1	19	2269485	2273487
OAZ2	15	64979773	64995480
OAZ3	1	151735445	151743806
OGFOD1	16	56485329	56511407
OGFOD2	12	123459250	123464588
OPLAH	8	145106167	145118929
OPRD1	1	29138654	29190208
OPRK1	8	54138276	54164257
OPRM1	6	154331631	154568001
ORM1	9	117085303	117088759
ORM2	9	117092069	117095536
OXT	20	3049517	3053163
OXTR	3	8792094	8896233
P2RY12	3	151054631	151102600
P2RY2	11	72927493	72953472
P4HA1	10	74766975	74856732
P4HB	17	79801034	79818544
P4HTM	3	49026533	49044582

PADI4	1	17634690	17690499
PAEP	9	138453600	138458622
PAH	12	103232099	103311381
PAICS	4	57301915	57327534
PAM	5	102090980	102366809
PANX1	11	93862094	93918762
PAPSS1	4	108534822	108641419
PCSK1	5	95726040	95768985
PCSK2	20	17206752	17465223
PCYT1A	3	195965253	196014584
PDXK	21	45138978	45182188
PF4	4	74846542	74847841
PFAS	17	8152573	8173809
PGF	14	75408533	75422467
PGR	11	100900355	101001399
PHOSPHO1	17	47300728	47309054
PIK3R1	5	67511584	67597649
PIK3R2	19	18263988	18281343
PIK3R3	1	46505812	46642167
PIPOX	17	27369918	27384236
PLA2G1B	12	120759914	120765592
PLA2G2E	1	20246800	20250110
PLA2G4A	1	186798032	186958113
PLAT	8	42032236	42065194
PLAU	10	75668940	75677259
PLAUR	19	44150247	44174498
PLD1	3	171318195	171528284
PLD2	17	4710396	4726727
PLEKHA1	10	124134094	124191871
PLG	6	161123225	161175086
PNLIP	10	118305428	118327367
PNP	14	20937538	20946165
POMC	2	25383722	25391740
PON1	7	94927669	94953884
PPARA	22	46546450	46639653
PPARD	6	35310335	35395968
PPARG	3	12329349	12512512
PRKAA1	5	40759481	40798297
PRKAB1	12	120105669	120119429
PRKCA	17	64298900	64806862
PRKCD	3	53195223	53226733
PRKDC	8	48685669	48872743

PRLR	5	35048861	35230691
PROC	2	128175981	128186822
PROCR	20	33759740	33803792
PROS1	3	93591881	93692934
PROZ	13	113809375	113826700
PRSS1	7	142448082	142460927
PSAT1	9	80911991	80945009
PTGDR	14	52733974	52743442
PTGER1	19	14583278	14586174
PTGER2	14	52781016	52795324
PTGER3	1	71318036	71513497
PTGER4	5	40679600	40741866
PTGFR	1	78941925	79006386
PTGIR	19	47113297	47128375
PTGIS	20	48120411	48184707
PTGR2	14	74318534	74352168
PTGS1	9	125132809	125157984
PTGS2	1	186640944	186649559
PTH1R	3	46919207	46945289
PTH2R	2	209224569	209359231
PTPN1	20	49126858	49201299
PTPN4	2	120517207	120742475
PTPRE	10	129705325	129884180
PTPRS	19	5205514	5340814
RAC1	7	6414126	6443598
RAC2	22	37621301	37640339
RAMP1	2	238768187	238820756
RAMP2	17	40913212	40915059
RAMP3	7	45197367	45223849
RARA	17	38465423	38513895
RARB	3	24870835	25162112
RARB	3	25215823	25639423
RARG	12	53604350	53626040
RARRES1	3	158414681	158450480
RB1	13	48877883	49056026
RBP1	3	139236276	139258671
REN	1	204123944	204135465
RHO	3	129247482	129254187
RHOB	2	20646832	20649206
RNASE1	14	21269515	21271036
RNASE3	14	21359562	21360507
RORB	9	77112252	77302117

RXRA	9	137218309	137332432
RXRB	6	33161362	33168630
RXRG	1	165370159	165414592
RYR1	19	38924340	39078204
S100A1	1	153600873	153604513
S100A12	1	153346184	153348075
S100A13	1	153591275	153606568
S100A2	1	153533584	153538306
S100A4	1	153516095	153518282
S100B	21	48018531	48025035
S100P	4	6695566	6698897
SCN10A	3	38738382	38835501
SCN11A	3	38887260	38995142
SCN1A	2	166845670	167005782
SCN1B	19	35521592	35531353
SCN2A	2	165986659	166248820
SCN2B	11	118033519	118047337
SCN3A	2	165944030	166060598
SCN3B	11	123499895	123525315
SCN4A	17	62015914	62067510
SCN4B	11	118004092	118023630
SCN5A	3	38589549	38691164
SCN7A	2	167259978	167350768
SCN8A	12	51983977	52206648
SCN9A	2	167051695	167232497
SCNN1A	12	6456009	6486523
SCNN1B	16	23313591	23392620
SCNN1D	1	1215795	1227409
SCNN1G	16	23194040	23228200
SELE	1	169691781	169703220
SELP	1	169558087	169599377
SERPINA5	14	95047706	95059457
SERPINB2	18	61554939	61571124
SERPINB6	6	2948393	2972399
SERPINC1	1	173872938	173886516
SERPIND1	22	21128383	21142008
SERPINE1	7	100770370	100782547
SHBG	17	7517382	7536701
SIGMAR1	9	34634719	34637823
SLC12A1	15	48498498	48596275
SLC12A2	5	127419483	127525380
SLC12A3	16	56899119	56949762

SLC12A4	16	67977377	68002597
SLC12A5	20	44650329	44688789
SLC15A1	13	99336055	99404929
SLC15A2	3	121613171	121663034
SLC18A1	8	20002366	20040849
SLC18A2	10	119000584	119038941
SLC19A3	2	228549926	228582745
SLC1A1	9	4490427	4587469
SLC1A2	11	35272752	35441610
SLC1A3	5	36606457	36688436
SLC1A6	19	15060845	15121455
SLC1A7	1	53552851	53608304
SLC22A11	11	64323098	64340347
SLC22A6	11	62744069	62752495
SLC22A7	6	43263448	43273276
SLC22A8	11	62760296	62783317
SLC23A1	5	138702885	138720242
SLC25A12	2	172639915	172750816
SLC25A13	7	95749532	95951459
SLC25A15	13	41363547	41386596
SLC25A18	22	18042865	18073656
SLC25A2	5	140682196	140683630
SLC25A22	11	790475	798269
SLC25A4	4	186064417	186071538
SLC29A1	6	44187242	44201888
SLC32A1	20	37353105	37358015
SLC36A1	5	150790905	150941522
SLC47A1	17	19437167	19482346
SLC52A2	8	145582217	145584946
SLC6A1	3	11034415	11080935
SLC6A2	16	55689516	55740104
SLC6A3	5	1392905	1445543
SLC6A4	17	28521337	28562986
SLC6A5	11	20620946	20680831
SLC6A9	1	44462155	44497164
SLC7A1	13	30083547	30169825
SLC7A11	4	139085248	139233814
SLC7A2	8	17354597	17428077
SLC7A4	22	21383007	21386847
SLC8A1	2	40339286	40739626
SLC9A1	1	27425300	27481621
SLCO1A2	12	21417534	21556591

SLCO1B1	12	21284128	21392730
SLCO1B3	12	20963638	21069845
SLCO2B1	11	74862032	74917445
SMO	7	128828713	128853387
SMPD1	11	6411531	6416228
SNAP25	20	10199477	10288068
SOAT1	1	179262849	179327815
SQLE	8	126010720	126034526
SRC	20	35973088	36033835
SRD5A1	5	6633473	6669675
SRD5A2	2	31747558	31925853
SSTR1	14	38677204	38682268
SSTR2	17	71161160	71168094
SSTR3	22	37600277	37616487
SSTR5	16	1122756	1131454
ST14	11	130029682	130080257
STAB2	12	103981033	104160507
SULT1E1	4	70706930	70726483
SV2A	1	149874870	149889434
TAAR1	6	132966123	132967142
TACR1	2	75273590	75426645
TAT	16	71600754	71610998
TBXAS1	7	139478047	139720125
TCN1	11	59620281	59634041
TEK	9	27109139	27230176
TERT	5	1253282	1295162
TF	3	133380842	133497850
TFPI	2	188328957	188419221
TGFB1	19	41836436	41859838
TGFBR2	3	30647985	30735634
TH	11	2185159	2193367
THBD	20	23026270	23030301
THRA	17	38218446	38250120
THRB	3	24158644	24536772
TLR9	3	52255096	52260179
TNF	6	31543344	31546113
TNFAIP6	2	152214097	152237759
TNFSF11	13	43136872	43182149
TNFSF13B	13	108921768	108960832
TOP1	20	39657462	39753127
TOP2A	17	38544773	38574202
TPMT	6	18128542	18155396

TPO	2	1417233	1546499
TRDMT1	10	17179801	17244070
TRPA1	8	72933486	73002244
TRPM8	2	234826007	234928166
TRPV1	17	3468740	3512705
TRPV3	17	3413796	3461289
TSPO	22	43547520	43559248
TUBA1A	12	49578578	49583107
TUBA4A	2	220114433	220119330
TUBB	6	30687978	30693203
TUBB1	20	57594309	57601709
TYMS	18	657604	673499
TYR	11	88911026	89028927
UCK2	1	165796732	165880855
UGT1A9	2	234580544	234681951
VCAM1	1	101185196	101204601
VCAN	5	82767493	82878122
VDR	12	48235320	48298814
VEGFA	6	43737946	43754224
VEGFB	11	64002056	64006736
VKORC1	16	31102175	31107658
VKORC1L1	7	65301323	65424550
VTN	17	26694298	26697373
VWF	12	6058040	6233836
XDH	2	31557175	31637615
AOC1	7	150523481	150558379
ASIC2	17	31340105	32483825
ASIC1	12	50451420	50477405
FECH	18	55212073	55253969
GOT1	10	101156627	101190530
MMP20	11	102447566	102496063
TUBB4B	9	140135711	140138159
CPQ	8	97657455	98155731
P3H3	12	6937538	6949018
PTGDR2	11	60618398	60623444
DBNL	7	44084239	44101315
P3H2	3	189674517	189840226
KYAT3	1	89401456	89458643
P3H1	1	43212006	43232755
MMP25	16	3095964	3110730
GADL1	3	30767692	30936153
GLCCI1	7	8008374	8128710

AZIN2	1	33546711	33644493

Supplementary Table 3: All ICD-9 codes used in the study along with their descriptions and categories

ICD-9_Code	ICD9_Description	ICD9_Category
110.1	Dermatophytosis_of_nail	Infectious_Diseases
110.4	Dermatophytosis_of_foot	Infectious_Diseases
110.5	Dermatophytosis_of_the_body	Infectious_Diseases
112	Candidiasis_of_mouth	Infectious_Diseases
112.1	Candidiasis_of_vulva_and_vagina	Infectious_Diseases
112.3	Candidiasis_of_skin_and_nails	Infectious_Diseases
135	Sarcoidosis	Infectious_Diseases
153.9	Malignant_neoplasm_of_colon,_unspecified_site	Neoplasms
162.3	Malignant_neoplasm_of_upper_lobe,_bronchus_or_lung	Neoplasms
162.9	Malignant_neoplasm_of_bronchus_and_lung,_unspecified	Neoplasms
173.3	Other_and_unspecified_malignant_neoplasm_of_skin_of_other_and_unspecified_parts_of_face	Neoplasms
174.4	Malignant_neoplasm_of_upper-outer_quadrant_of_female_breast	Neoplasms
174.9	Malignant_neoplasm_of_breast_(female),_unspecified	Neoplasms
188.9	Malignant_neoplasm_of_bladder,_part_unspecified	Neoplasms
189	Malignant_neoplasm_of_kidney,_except_pelvis	Neoplasms
193	Malignant_neoplasm_of_thyroid_gland	Neoplasms
202.8	Other_malignant_lymphomas,_unspecified_site,_extranodal_and_solid_organ_sites	Neoplasms
211.3	Benign_neoplasm_of_colon	Neoplasms
216.9	Benign_neoplasm_of_skin,_site_unspecified	Neoplasms
227.3	Benign_neoplasm_of_pituitary_gland_and_craniopharyngeal_duct	Neoplasms
238.2	Neoplasm_of_uncertain_behavior_of_skin	Neoplasms
238.9	Neoplasm_of_uncertain_behavior,_site_unspecified	Neoplasms
240.9	Goiter,_unspecified	Endocrine_and_Metabolic
241	Nontoxic_uninodular_goiter	Endocrine_and_Metabolic

241.1	Nontoxic_multinodular_goiter	Endocrine_and_Metabolic
242	Toxic_diffuse_goiter_without_mention_of_thyrotoxic_crisis_or_storm	Endocrine_and_Metabolic
242.9	Thyrotoxicosis_without_mention_of_goiter_or_other_cause,_and_without_mention_of_thyrotoxic_crisis_or_storm	Endocrine_and_Metabolic
244	Postsurgical_hypothyroidism	Endocrine_and_Metabolic
244.1	Other_postablative_hypothyroidism	Endocrine_and_Metabolic
244.8	Other_specified_acquired_hypothyroidism	Endocrine_and_Metabolic
244.9	Unspecified_acquired_hypothyroidism	Endocrine_and_Metabolic
245.2	Chronic_lymphocytic_thyroiditis	Endocrine_and_Metabolic
250	Diabetes_mellitus_without_mention_of_complication,_type_II_or_unspecified_type,_not_stated_as_uncontrolled	Endocrine_and_Metabolic
250.01	Diabetes_mellitus_without_mention_of_complication,_type_I_[juvenile_type],_not_stated_as_uncontrolled	Endocrine_and_Metabolic
250.4	Diabetes_with_renal_manifestations,_type_II_or_unspecified_type,_not_stated_as_uncontrolled	Endocrine_and_Metabolic
250.5	Diabetes_with_ophthalmic_manifestations,_type_II_or_unspecified_type,_not_stated_as_uncontrolled	Endocrine_and_Metabolic
250.6	Diabetes_with_neurological_manifestations,_type_II_or_unspecified_type,_not_stated_as_uncontrolled	Endocrine_and_Metabolic
250.7	Diabetes_with_peripheral_circulatory_disorders,_type_II_or_unspecified_type,_not_stated_as_uncontrolled	Endocrine_and_Metabolic
250.8	Diabetes_with_other_specified_manifestations,_type_II_or_unspecified_type,_not_stated_as_uncontrolled	Endocrine_and_Metabolic
251.1	Other_specified_hypoglycemia	Endocrine_and_Metabolic
251.2	Hypoglycemia,_unspecified	Endocrine_and_Metabolic
252.01	Primary_hyperparathyroidism	Endocrine_and_Metabolic
266.2	Other_B-complex_deficiencies	Endocrine_and_Metabolic
268.9	Unspecified_vitamin_D_deficiency	Endocrine_and_Metabolic
269.2	Unspecified_vitamin_deficiency	Endocrine_and_Metabolic

272	Pure_hypercholesterolemia	Endocrine_and_Metabolic
272.1	Pure_hyperglyceridemia	Endocrine_and_Metabolic
272.2	Mixed_hyperlipidemia	Endocrine_and_Metabolic
272.4	Other_and_unspecified_hyperlipidemia	Endocrine_and_Metabolic
272.5	Lipoprotein_deficiencies	Endocrine_and_Metabolic
273.1	Monoclonal_paraproteinemia	Endocrine_and_Metabolic
274	Gouty_arthropathy	Endocrine_and_Metabolic
274	Gouty_arthropathy,_unspecified	Endocrine_and_Metabolic
274.9	Gout,_unspecified	Endocrine_and_Metabolic
275.42	Hypercalcemia	Endocrine_and_Metabolic
276.1	Hyposmolality_and/or_hyponatremia	Endocrine_and_Metabolic
276.51	Dehydration	Endocrine_and_Metabolic
276.6	Fluid_overload	Endocrine_and_Metabolic
276.7	Hyperpotassemia	Endocrine_and_Metabolic
276.8	Hypopotassemia	Endocrine_and_Metabolic
276.9	Electrolyte_and_fluid_disorders_not_elsewhere_classified	Endocrine_and_Metabolic
277.7	Dysmetabolic_syndrome_X	Endocrine_and_Metabolic
278	Obesity,_unspecified	Endocrine_and_Metabolic
278.01	Morbid_obesity	Endocrine_and_Metabolic
278.02	Overweight	Endocrine_and_Metabolic
278.1	Localized_adiposity	Endocrine_and_Metabolic
280.9	Iron_deficiency_anemia,_unspecified	Blood_Disorder
281	Pernicious_anemia	Blood_Disorder
281.1	Other_vitamin_B12_deficiency_anemia	Blood_Disorder
285.1	Acute_posthemorrhagic_anemia	Blood_Disorder
285.21	Anemia_in_chronic_kidney_disease	Blood_Disorder
285.9	Anemia,_unspecified	Blood_Disorder
287.5	Thrombocytopenia,_unspecified	Blood_Disorder
289.81	Primary_hypercoagulable_state	Blood_Disorder

296.2	Major_depressive_affective_disorder,_single_episode,_unspecified	Mental_Disorders
296.22	Major_depressive_affective_disorder,_single_episode,_moderate	Mental_Disorders
296.3	Major_depressive_affective_disorder,_recurrent_episode,_unspecified	Mental_Disorders
296.32	Major_depressive_affective_disorder,_recurrent_episode,_moderate	Mental_Disorders
296.8	Bipolar_disorder,_unspecified	Mental_Disorders
296.9	Unspecified_episodic_mood_disorder	Mental_Disorders
300	Anxiety_state,_unspecified	Mental_Disorders
300.01	Panic_disorder_without_agoraphobia	Mental_Disorders
300.02	Generalized_anxiety_disorder	Mental_Disorders
300.09	Other_anxiety_states	Mental_Disorders
300.4	Dysthymic_disorder	Mental_Disorders
305	Alcohol_abuse,_unspecified	Mental_Disorders
305.1	Tobacco_use_disorder	Mental_Disorders
307.42	Persistent_disorder_of_initiating_or_maintaining_sleep	Mental_Disorders
307.5	Eating_disorder,_unspecified	Mental_Disorders
307.81	Tension_headache	Mental_Disorders
309	Adjustment_disorder_with_depressed_mood	Mental_Disorders
309.24	Adjustment_disorder_with_anxiety	Mental_Disorders
309.28	Adjustment_disorder_with_mixed_anxiety_and_depressed_mood	Mental_Disorders
311	Depressive_disorder,_not_elsewhere_classified	Mental_Disorders
314	Attention_deficit_disorder_without_mention_of_hyperactivity	Mental_Disorders
314.01	Attention_deficit_disorder_with_hyperactivity	Mental_Disorders
327.23	Obstructive_sleep_apnea_(adult)(pediatric)	Nervous_System
327.8	Other_organic_sleep_disorders	Nervous_System
332	Paralysis_agitans	Nervous_System
333.1	Essential_and_other_specified_forms_of_tremor	Nervous_System
333.94	Restless_legs_syndrome_(RLS)	Nervous_System
338.29	Other_chronic_pain	Nervous_System
338.4	Chronic_pain_syndrome	Nervous_System

340	Multiple_sclerosis	Nervous_System
345.1	Generalized_convulsive_epilepsy,_without_mention_of_intractable_epilepsy	Nervous_System
345.9	Epilepsy,_unspecified,_without_mention_of_intractable_epilepsy	Nervous_System
346	Migraine_with_aura,_without_mention_of_intractable_migraine_without_mention_of_status_migrainosus	Nervous_System
346.01	Migraine_with_aura,_with_intractable_migraine,_so_stated,_without_mention_of_status_migrainosus	Nervous_System
346.1	Migraine_without_aura,_without_mention_of_intractable_migraine_without_mention_of_status_migrainosus	Nervous_System
346.11	Migraine_without_aura,_with_intractable_migraine,_so_stated,_without_mention_of_status_migrainosus	Nervous_System
346.2	Variants_of_migraine,_not_elsewhere_classified,_without_mention_of_intractable_migraine_without_mention_of_status_migrainosus	Nervous_System
346.9	Migraine,_unspecified,_without_mention_of_intractable_migraine_without_mention_of_status_migrainosus	Nervous_System
354	Carpal_tunnel_syndrome	Nervous_System
354.2	Lesion_of_ulnar_nerve	Nervous_System
355.9	Mononeuritis_of_unspecified_site	Nervous_System
356.9	Unspecified_hereditary_and_idiopathic_peripheral_neuropathy	Nervous_System
357.2	Polyneuropathy_in_diabetes	Nervous_System
357.4	Polyneuropathy_in_other_diseases_classified_elsewhere	Nervous_System
361	Retinal_detachment_with_retinal_defect,_unspecified	Nervous_System
362.01	Background_diabetic_retinopathy	Nervous_System
362.02	Proliferative_diabetic_retinopathy	Nervous_System
362.07	Diabetic_macular_edema	Nervous_System
362.5	Macular_degeneration_(senile),_unspecified	Nervous_System
362.51	Nonexudative_senile_macular_degeneration	Nervous_System
362.52	Exudative_senile_macular_degeneration	Nervous_System
362.56	Macular_puckering	Nervous_System

362.83	Retinal_edema	Nervous_System
364.3	Unspecified_iridocyclitis	Nervous_System
365	Preglaucoma,_unspecified	Nervous_System
365.02	Anatomical_narrow_angle_borderline_glaucoma	Nervous_System
365.04	Ocular_hypertension	Nervous_System
365.11	Primary_open_angle_glaucoma	Nervous_System
365.2	Primary_angle-closure_glaucoma,_unspecified	Nervous_System
365.7	Glaucoma_stage,_unspecified	Nervous_System
365.9	Unspecified_glaucoma	Nervous_System
366.1	Senile_cataract,_unspecified	Nervous_System
366.12	Incipient_senile_cataract	Nervous_System
366.14	Posterior_subcapsular_polar_senile_cataract	Nervous_System
366.15	Cortical_senile_cataract	Nervous_System
366.16	Senile_nuclear_sclerosis	Nervous_System
366.52	Other_after-cataract,_not_obscuring_vision	Nervous_System
366.53	After-cataract,_obscuring_vision	Nervous_System
366.9	Unspecified_cataract	Nervous_System
367.4	Presbyopia	Nervous_System
367.9	Unspecified_disorder_of_refraction_and_accommodation	Nervous_System
368.2	Diplopia	Nervous_System
370.33	Keratoconjunctivitis_sicca,_not_specified_as_Sjogren's	Nervous_System
371.52	Other_anterior_corneal_dystrophies	Nervous_System
371.57	Endothelial_corneal_dystrophy	Nervous_System
372.14	Other_chronic_allergic_conjunctivitis	Nervous_System
372.3	Conjunctivitis,_unspecified	Nervous_System
373	Blepharitis,_unspecified	Nervous_System
374.3	Ptosis_of_eyelid,_unspecified	Nervous_System
374.87	Dermatochalasis	Nervous_System
375.15	Tear_film_insufficiency,_unspecified	Nervous_System

379.21	Vitreous_degeneration	Nervous_System
379.23	Vitreous_hemorrhage	Nervous_System
38.9	Unspecified_septicemia	Infectious_Diseases
380.1	Infective_otitis_externa,_unspecified	Nervous_System
380.22	Other_acute_otitis_externa	Nervous_System
380.4	Impacted_cerumen	Nervous_System
381.01	Acute_serous_otitis_media	Nervous_System
381.81	Dysfunction_of_Eustachian_tube	Nervous_System
382.9	Unspecified_otitis_media	Nervous_System
386.11	Benign_paroxysmal_positional_vertigo	Nervous_System
388.7	Otalgia,_unspecified	Nervous_System
389.1	Sensorineural_hearing_loss,_unspecified	Nervous_System
389.9	Unspecified_hearing_loss	Nervous_System
397	Diseases_of_tricuspid_valve	Circulatory
401.9	Unspecified_essential_hypertension	Circulatory
402.9	Unspecified_hypertensive_heart_disease_without_heart_failure	Circulatory
402.91	Unspecified_hypertensive_heart_disease_with_heart_failure	Circulatory
403.9	Hypertensive_chronic_kidney_disease,_unspecified,_with_chronic_kidney_disease_ stage_I_through_stage_IV,_or_unspecified	Circulatory
403.91	Hypertensive_chronic_kidney_disease,_unspecified,_with_chronic_kidney_disease_ stage_V_or_end_stage_renal_disease	Circulatory
410.7	Subendocardial_infarction,_episode_of_care_unspecified	Circulatory
410.9	Acute_myocardial_infarction_of_unspecified_site,_episode_of_care_unspecified	Circulatory
411.1	Intermediate_coronary_syndrome	Circulatory
412	Old_myocardial_infarction	Circulatory
413.9	Other_and_unspecified_angina_pectoris	Circulatory
414	Coronary_atherosclerosis_of_unspecified_type_of_vessel,_native_or_graft	Circulatory
414.01	Coronary_atherosclerosis_of_native_coronary_artery	Circulatory
414.8	Other_specified_forms_of_chronic_ischemic_heart_disease	Circulatory

414.9	Chronic_ischemic_heart_disease,_unspecified	Circulatory
415.19	Other_pulmonary_embolism_and_infarction	Circulatory
416.8	Other_chronic_pulmonary_heart_diseases	Circulatory
424	Mitral_valve_disorders	Circulatory
424.1	Aortic_valve_disorders	Circulatory
424.9	Endocarditis,_valve_unspecified,_unspecified_cause	Circulatory
425.4	Other_primary_cardiomyopathies	Circulatory
426	Atrioventricular_block,_complete	Circulatory
426.3	Other_left_bundle_branch_block	Circulatory
427	Paroxysmal_supraventricular_tachycardia	Circulatory
427.1	Paroxysmal_ventricular_tachycardia	Circulatory
427.31	Atrial_fibrillation	Circulatory
427.32	Atrial_flutter	Circulatory
427.69	Other_premature_beats	Circulatory
427.81	Sinoatrial_node_dysfunction	Circulatory
427.89	Other_specified_cardiac_dysrhythmias	Circulatory
428	Congestive_heart_failure,_unspecified	Circulatory
428.2	Systolic_heart_failure,_unspecified	Circulatory
428.22	Chronic_systolic_heart_failure	Circulatory
428.23	Acute_on_chronic_systolic_heart_failure	Circulatory
428.3	Diastolic_heart_failure,_unspecified	Circulatory
428.32	Chronic_diastolic_heart_failure	Circulatory
428.33	Acute_on_chronic_diastolic_heart_failure	Circulatory
428.9	Heart_failure,_unspecified	Circulatory
429.2	Cardiovascular_disease,_unspecified	Circulatory
429.9	Heart_disease,_unspecified	Circulatory
433.1	Occlusion_and_stenosis_of_carotid_artery_without_mention_of_cerebral_infarction	Circulatory
433.11	Occlusion_and_stenosis_of_carotid_artery_with_cerebral_infarction	Circulatory

434.91	Cerebral_artery_occlusion,_unspecified_with_cerebral_infarction	Circulatory
435.9	Unspecified_transient_cerebral_ischemia	Circulatory
436	Acute,_but_ill-defined,_cerebrovascular_disease	Circulatory
437	Cerebral_atherosclerosis	Circulatory
437.3	Cerebral_aneurysm,_nonruptured	Circulatory
440.1	Atherosclerosis_of_renal_artery	Circulatory
440.2	Atherosclerosis_of_native_arteries_of_the_extremities,_unspecified	Circulatory
440.21	Atherosclerosis_of_native_arteries_of_the_extremities_with_intermittent_claudica tion	Circulatory
440.23	Atherosclerosis_of_native_arteries_of_the_extremities_with_ulceration	Circulatory
440.9	Generalized_and_unspecified_atherosclerosis	Circulatory
441.2	Thoracic_aneurysm_without_mention_of_rupture	Circulatory
441.4	Abdominal_aneurysm_without_mention_of_rupture	Circulatory
443	Raynaud's_syndrome	Circulatory
443.81	Peripheral_angiopathy_in_diseases_classified_elsewhere	Circulatory
443.9	Peripheral_vascular_disease,_unspecified	Circulatory
451.19	Phlebitis_and_thrombophlebitis_of_deep_veins_of_lower_extremities,_other	Circulatory
453.4	Acute_venous_embolism_and_thrombosis_of_unspecified_deep_vessels_of_lower _extremity	Circulatory
453.9	Other_venous_embolism_and_thrombosis_of_unspecified_site	Circulatory
454	Varicose_veins_of_lower_extremities_with_ulcer	Circulatory
454.8	Varicose_veins_of_lower_extremities_with_other_complications	Circulatory
454.9	Asymptomatic_varicose_veins	Circulatory
455.3	External_hemorrhoids_without_mention_of_complication	Circulatory
455.6	Unspecified_hemorrhoids_without_mention_of_complication	Circulatory
458	Orthostatic_hypotension	Circulatory
458.9	Hypotension,_unspecified	Circulatory
459.81	Venous_(peripheral)_insufficiency,_unspecified	Circulatory
461	Acute_maxillary_sinusitis	Respiratory

461.9	Acute_sinusitis,_unspecified	Respiratory
462	Acute_pharyngitis	Respiratory
465.9	Acute_upper_respiratory_infections_of_unspecified_site	Respiratory
466	Acute_bronchitis	Respiratory
470	Deviated_nasal_septum	Respiratory
472	Chronic_rhinitis	Respiratory
473.8	Other_chronic_sinusitis	Respiratory
473.9	Unspecified_sinusitis_(chronic)	Respiratory
477.8	Allergic_rhinitis_due_to_other_allergen	Respiratory
477.9	Allergic_rhinitis,_cause_unspecified	Respiratory
482.9	Bacterial_pneumonia,_unspecified	Respiratory
486	Pneumonia,_organism_unspecified	Respiratory
490	Bronchitis,_not_specified_as_acute_or_chronic	Respiratory
491.21	Obstructive_chronic_bronchitis_with_(acute)_exacerbation	Respiratory
492.8	Other_emphysema	Respiratory
493.2	Chronic_obstructive_asthma,_unspecified	Respiratory
493.9	Asthma,_unspecified_type,_unspecified	Respiratory
493.92	Asthma,_unspecified_type,_with_(acute)_exacerbation	Respiratory
496	Chronic_airway_obstruction,_not_elsewhere_classified	Respiratory
511.9	Unspecified_pleural_effusion	Respiratory
515	Postinflammatory_pulmonary_fibrosis	Respiratory
518.89	Other_diseases_of_lung,_not_elsewhere_classified	Respiratory
520.6	Disturbances_in_tooth_eruption	Digestive
521	Dental_caries	Digestive
521	Dental_caries,_unspecified	Digestive
521.02	Dental_caries_extending_into_dentine	Digestive
522.5	Periapical_abscess_without_sinus	Digestive
524.6	Temporomandibular_joint_disorders,_unspecified	Digestive
525.1	Loss_of_teeth_due_to_trauma,_extraction,_or_periodontal_disease	Digestive

53.9	Herpes_zoster_without_mention_of_complication	Infectious_Diseases
530.1	Esophagitis,_unspecified	Digestive
530.11	Reflux_esophagitis	Digestive
530.81	Esophageal_reflux	Digestive
530.85	Barrett's_esophagus	Digestive
533.9	Peptic_ulcer_of_unspecified_site,_unspecified_as_acute_or_chronic,_without_mention_of_hemorrhage_or_perforation,_without_mention_of_obstruction	Digestive
535.5	Unspecified_gastritis_and_gastroduodenitis,_without_mention_of_hemorrhage	Digestive
536.3	Gastroparesis	Digestive
54.9	Herpes_simplex_without_mention_of_complication	Infectious_Diseases
550.9	Inguinal_hernia,_without_mention_of_obstruction_or_gangrene,_unilateral_or_unspecified_(not_specified_as_recurrent)	Digestive
553.1	Umbilical_hernia_without_mention_of_obstruction_or_gangrene	Digestive
553.2	Ventral,_unspecified,_hernia_without_mention_of_obstruction_or_gangrene	Digestive
553.21	Incisional_hernia_without_mention_of_obstruction_or_gangrene	Digestive
553.3	Diaphragmatic_hernia_without_mention_of_obstruction_or_gangrene	Digestive
555.9	Regional_enteritis_of_unspecified_site	Digestive
556.9	Ulcerative_colitis,_unspecified	Digestive
560.9	Unspecified_intestinal_obstruction	Digestive
562.1	Diverticulosis_of_colon_(without_mention_of_hemorrhage)	Digestive
562.11	Diverticulitis_of_colon_(without_mention_of_hemorrhage)	Digestive
564	Constipation,_unspecified	Digestive
564.09	Other_constipation	Digestive
564.1	Irritable_bowel_syndrome	Digestive
564.2	Postgastric_surgery_syndromes	Digestive
569.3	Hemorrhage_of_rectum_and_anus	Digestive
571.5	Cirrhosis_of_liver_without_mention_of_alcohol	Digestive
571.8	Other_chronic_nonalcoholic_liver_disease	Digestive

574.2	Calculus_of_gallbladder_without_mention_of_cholecystitis,_without_mention_of_obstruction	Digestive
577	Acute_pancreatitis	Digestive
578.9	Hemorrhage_of_gastrointestinal_tract,_unspecified	Digestive
579.3	Other_and_unspecified_postsurgical_nonabsorption	Digestive
583.81	Nephritis_and_nephropathy,_not_specified_as_acute_or_chronic,_in_diseases_classified_elsewhere	Genitourinary
584.9	Acute_kidney_failure,_unspecified	Genitourinary
585.3	Chronic_kidney_disease,_Stage_III_(moderate)	Genitourinary
585.4	Chronic_kidney_disease,_Stage_IV_(severe)	Genitourinary
585.5	Chronic_kidney_disease,_Stage_V	Genitourinary
585.6	End_stage_renal_disease	Genitourinary
585.9	Chronic_kidney_disease,_unspecified	Genitourinary
586	Renal_failure,_unspecified	Genitourinary
591	Hydronephrosis	Genitourinary
592	Calculus_of_kidney	Genitourinary
592.1	Calculus_of_ureter	Genitourinary
593.2	Cyst_of_kidney,_acquired	Genitourinary
593.9	Unspecified_disorder_of_kidney_and_ureter	Genitourinary
595	Acute_cystitis	Genitourinary
596.54	Neurogenic_bladder_NOS	Genitourinary
599	Urinary_tract_infection,_site_not_specified	Genitourinary
599.7	Hematuria	Genitourinary
599.7	Hematuria,_unspecified	Genitourinary
599.71	Gross_hematuria	Genitourinary
607.84	Impotence_of_organic_origin	Genitourinary
611.1	Hypertrophy_of_breast	Genitourinary
611.72	Lump_or_mass_in_breast	Genitourinary
618.01	Cystocele,_midline	Genitourinary

625.9	Unspecified_symptom_associated_with_female_genital_organs	Genitourinary
627.2	Symptomatic_menopausal_or_female_climacteric_states	Genitourinary
648.83	Abnormal_glucose_tolerance_of_mother,_antepartum_condition_or_complication	Pregnancy_related
682.2	Cellulitis_and_abscess_of_trunk	Skin
682.6	Cellulitis_and_abscess_of_leg,_except_foot	Skin
682.7	Cellulitis_and_abscess_of_foot,_except_toes	Skin
682.9	Cellulitis_and_abscess_of_unspecified_sites	Skin
690.1	Seborrheic_dermatitis,_unspecified	Skin
692.6	Contact_dermatitis_and_other_eczema_due_to_plants_[except_food]	Skin
692.9	Contact_dermatitis_and_other_eczema,_unspecified_cause	Skin
695.3	Rosacea	Skin
695.89	Other_specified_erythematous_conditions	Skin
696	Psoriatic_arthropathy	Skin
696.1	Other_psoriasis	Skin
698.9	Unspecified_pruritic_disorder	Skin
70.54	Chronic_hepatitis_C_without_mention_of_hepatic_coma	Infectious_Diseases
700	Corns_and_callosities	Skin
701.9	Unspecified_hypertrophic_and_atrophic_conditions_of_skin	Skin
702	Actinic_keratosis	Skin
702.11	Inflamed_seborrheic_keratosis	Skin
702.19	Other_seborrheic_keratosis	Skin
703	Ingrowing_nail	Skin
703.8	Other_specified_diseases_of_nail	Skin
704.1	Hirsutism	Skin
704.8	Other_specified_diseases_of_hair_and_hair_follicles	Skin
706.1	Other_acne	Skin
706.2	Sebaceous_cyst	Skin
707.1	Ulcer_of_lower_limb,_unspecified	Skin
707.14	Ulcer_of_heel_and_midfoot	Skin

707.15	Ulcer_of_other_part_of_foot	Skin
707.9	Chronic_ulcer_of_unspecified_site	Skin
708.9	Urticaria,_unspecified	Skin
709.2	Scar_conditions_and_fibrosis_of_skin	Skin
709.9	Unspecified_disorder_of_skin_and_subcutaneous_tissue	Skin
710	Systemic_lupus_erythematosus	Musculoskeletal
710.2	Sicca_syndrome	Musculoskeletal
714	Rheumatoid_arthritis	Musculoskeletal
715	Osteoarthrosis,_generalized,_site_unspecified	Musculoskeletal
715.09	Osteoarthrosis,_generalized,_multiple_sites	Musculoskeletal
715.16	Osteoarthrosis,_localized,_primary,_lower_leg	Musculoskeletal
715.34	Osteoarthrosis,_localized,_not_specified_whether_primary_or_secondary,_hand	Musculoskeletal
715.9	Osteoarthrosis,_unspecified_whether_generalized_or_localized,_site_unspecified	Musculoskeletal
715.91	Osteoarthrosis,_unspecified_whether_generalized_or_localized,_shoulder_region	Musculoskeletal
715.94	Osteoarthrosis,_unspecified_whether_generalized_or_localized,_hand	Musculoskeletal
715.95	Osteoarthrosis,_unspecified_whether_generalized_or_localized,_pelvic_region_and _thigh	Musculoskeletal
715.96	Osteoarthrosis,_unspecified_whether_generalized_or_localized,_lower_leg	Musculoskeletal
715.98	Osteoarthrosis,_unspecified_whether_generalized_or_localized,_other_specified_si tes	Musculoskeletal
716.59	Unspecified_polyarthropathy_or_polyarthritis,_multiple_sites	Musculoskeletal
716.96	Arthropathy,_unspecified,_lower_leg	Musculoskeletal
717.9	Unspecified_internal_derangement_of_knee	Musculoskeletal
719.41	Pain_in_joint,_shoulder_region	Musculoskeletal
719.43	Pain_in_joint,_forearm	Musculoskeletal
719.44	Pain_in_joint,_hand	Musculoskeletal
719.45	Pain_in_joint,_pelvic_region_and_thigh	Musculoskeletal
719.46	Pain_in_joint,_lower_leg	Musculoskeletal
719.47	Pain_in_joint,_ankle_and_foot	Musculoskeletal

719.49	Pain_in_joint,_multiple_sites	Musculoskeletal
719.7	Difficulty_in_walking	Musculoskeletal
720.2	Sacroiliitis,_not_elsewhere_classified	Musculoskeletal
721	Cervical_spondylosis_without_myelopathy	Musculoskeletal
721.3	Lumbosacral_spondylosis_without_myelopathy	Musculoskeletal
722	Displacement_of_cervical_intervertebral_disc_without_myelopathy	Musculoskeletal
722.1	Displacement_of_lumbar_intervertebral_disc_without_myelopathy	Musculoskeletal
722.4	Degeneration_of_cervical_intervertebral_disc	Musculoskeletal
722.52	Degeneration_of_lumbar_or_lumbosacral_intervertebral_disc	Musculoskeletal
722.6	Degeneration_of_intervertebral_disc,_site_unspecified	Musculoskeletal
722.83	Postlaminectomy_syndrome,_lumbar_region	Musculoskeletal
722.9	Other_and_unspecified_disc_disorder,_unspecified_region	Musculoskeletal
722.91	Other_and_unspecified_disc_disorder,_cervical_region	Musculoskeletal
722.93	Other_and_unspecified_disc_disorder,_lumbar_region	Musculoskeletal
723	Spinal_stenosis_in_cervical_region	Musculoskeletal
723.1	Cervicalgia	Musculoskeletal
723.4	Brachial_neuritis_or_radiculitis_NOS	Musculoskeletal
723.8	Other_syndromes_affecting_cervical_region	Musculoskeletal
724	Spinal_stenosis,_unspecified_region	Musculoskeletal
724.02	Spinal_stenosis,_lumbar_region,_without_neurogenic_claudication	Musculoskeletal
724.1	Pain_in_thoracic_spine	Musculoskeletal
724.2	Lumbago	Musculoskeletal
724.3	Sciatica	Musculoskeletal
724.4	Thoracic_or_lumbosacral_neuritis_or_radiculitis,_unspecified	Musculoskeletal
724.5	Backache,_unspecified	Musculoskeletal
725	Polymyalgia_rheumatica	Musculoskeletal
726	Adhesive_capsulitis_of_shoulder	Musculoskeletal
726.1	Disorders_of_bursae_and_tendons_in_shoulder_region,_unspecified	Musculoskeletal
726.2	Other_affections_of_shoulder_region,_not_elsewhere_classified	Musculoskeletal

726.32	Lateral_epicondylitis	Musculoskeletal
726.33	Olecranon_bursitis	Musculoskeletal
726.5	Enthesopathy_of_hip_region	Musculoskeletal
726.6	Enthesopathy_of_knee,_unspecified	Musculoskeletal
726.71	Achilles_bursitis_or_tendinitis	Musculoskeletal
727.03	Trigger_finger_(acquired)	Musculoskeletal
727.05	Other_tenosynovitis_of_hand_and_wrist	Musculoskeletal
727.1	Bunion	Musculoskeletal
728.71	Plantar_fascial_fibromatosis	Musculoskeletal
728.85	Spasm_of_muscle	Musculoskeletal
729.1	Myalgia_and_myositis,_unspecified	Musculoskeletal
729.2	Neuralgia,_neuritis,_and_radiculitis,_unspecified	Musculoskeletal
729.3	Panniculitis,_unspecified_site	Musculoskeletal
729.5	Pain_in_limb	Musculoskeletal
729.82	Cramp_of_limb	Musculoskeletal
733	Osteoporosis,_unspecified	Musculoskeletal
733.01	Senile_osteoporosis	Musculoskeletal
733.09	Other_osteoporosis	Musculoskeletal
733.13	Pathologic_fracture_of_vertebrae	Musculoskeletal
733.9	Disorder_of_bone_and_cartilage,_unspecified	Musculoskeletal
737.3	Scoliosis_[and_kyphoscoliosis],_idiopathic	Musculoskeletal
739.1	Nonallopathic_lesions,_cervical_region	Musculoskeletal
739.2	Nonallopathic_lesions,_thoracic_region	Musculoskeletal
739.3	Nonallopathic_lesions,_lumbar_region	Musculoskeletal
745.5	Ostium_secundum_type_atrial_septal_defect	Congenital_Anomalies
756.12	Spondylolisthesis	Congenital_Anomalies
78.1	Viral_warts,_unspecified	Infectious_Diseases
78.19	Other_specified_viral_warts	Infectious_Diseases
780.2	Syncope_and_collapse	Symptoms

780.39	Other_convulsions	Symptoms
780.4	Dizziness_and_giddiness	Symptoms
780.52	Insomnia,_unspecified	Symptoms
780.53	Hypersomnia_with_sleep_apnea,_unspecified	Symptoms
780.57	Unspecified_sleep_apnea	Symptoms
780.6	Fever,_unspecified	Symptoms
780.79	Other_malaise_and_fatigue	Symptoms
780.93	Memory_loss	Symptoms
781	Abnormal_involuntary_movements	Symptoms
781.2	Abnormality_of_gait	Symptoms
782	Disturbance_of_skin_sensation	Symptoms
782.1	Rash_and_other_nonspecific_skin_eruption	Symptoms
782.3	Edema	Symptoms
783.1	Abnormal_weight_gain	Symptoms
783.21	Loss_of_weight	Symptoms
784	Headache	Symptoms
784.2	Swelling,_mass,_or_lump_in_head_and_neck	Symptoms
784.7	Epistaxis	Symptoms
785	Tachycardia,_unspecified	Symptoms
785.1	Palpitations	Symptoms
785.2	Undiagnosed_cardiac_murmurs	Symptoms
785.6	Enlargement_of_lymph_nodes	Symptoms
785.9	Other_symptoms_involving_cardiovascular_system	Symptoms
786.05	Shortness_of_breath	Symptoms
786.07	Wheezing	Symptoms
786.09	Other_respiratory_abnormalities	Symptoms
786.2	Cough	Symptoms
786.5	Chest_pain,_unspecified	Symptoms
786.59	Other_chest_pain	Symptoms

786.6	Swelling,_mass,_or_lump_in_chest	Symptoms
786.9	Other_symptoms_involving_respiratory_system_and_chest	Symptoms
787.01	Nausea_with_vomiting	Symptoms
787.02	Nausea_alone	Symptoms
787.1	Heartburn	Symptoms
787.2	Dysphagia	Symptoms
787.2	Dysphagia,_unspecified	Symptoms
787.91	Diarrhea	Symptoms
788.1	Dysuria	Symptoms
788.2	Retention_of_urine,_unspecified	Symptoms
788.21	Incomplete_bladder_emptying	Symptoms
788.3	Urinary_incontinence,_unspecified	Symptoms
788.31	Urge_incontinence	Symptoms
788.33	Mixed_incontinence_(male)_(female)	Symptoms
788.41	Urinary_frequency	Symptoms
788.43	Nocturia	Symptoms
788.63	Urgency_of_urination	Symptoms
789	Abdominal_pain,_unspecified_site	Symptoms
789.01	Abdominal_pain,_right_upper_quadrant	Symptoms
789.03	Abdominal_pain,_right_lower_quadrant	Symptoms
789.04	Abdominal_pain,_left_lower_quadrant	Symptoms
789.06	Abdominal_pain,_epigastric	Symptoms
789.07	Abdominal_pain,_generalized	Symptoms
789.09	Abdominal_pain,_other_specified_site	Symptoms
79.99	Unspecified_viral_infection	Infectious_Diseases
790.21	Impaired_fasting_glucose	Symptoms
790.29	Other_abnormal_glucose	Symptoms
790.4	Nonspecific_elevation_of_levels_of_transaminase_or_lactic_acid_dehydrogenase_[LDH]	Symptoms

790.5	Other_nonspecific_abnormal_serum_enzyme_levels	Symptoms
790.6	Other_abnormal_blood_chemistry	Symptoms
791	Proteinuria	Symptoms
793.11	Solitary_pulmonary_nodule	Symptoms
794.31	Nonspecific_abnormal_electrocardiogram_[ECG]_[EKG]	Symptoms
794.39	Other_nonspecific_abnormal_results_of_function_study_of_cardiovascular_system	Symptoms
794.8	Nonspecific_abnormal_results_of_function_study_of_liver	Symptoms
795.79	Other_and_unspecified_nonspecific_immunological_findings	Symptoms
796.2	Elevated_blood_pressure_reading_without_diagnosis_of_hypertension	Symptoms
799.02	Hypoxemia	Symptoms
8.45	Intestinal_infection_due_to_Clostridium_difficile	Infectious_Diseases
813.42	Other_closed_fractures_of_distal_end_of_radius_(alone)	Injury_And_Poisoning
824.8	Unspecified_fracture_of_ankle,_closed	Injury_And_Poisoning
825.25	Closed_fracture_of_metatarsal_bone(s)	Injury_And_Poisoning
840.4	Rotator_cuff_(capsule)_sprain	Injury_And_Poisoning
845	Sprain_of_ankle,_unspecified_site	Injury_And_Poisoning
846	Sprain_of_lumbosacral_(joint)_(ligament)	Injury_And_Poisoning
847	Sprain_of_neck	Injury_And_Poisoning
873.63	Open_wound_of_tooth_(broken)_(fractured)_(due_to_trauma),_without_mention_of_complication	Injury_And_Poisoning
891	Open_wound_of_knee,_leg_[except_thigh],_and_ankle,_without_mention_of_complication	Injury_And_Poisoning
995.3	Allergy,_unspecified,_not_elsewhere_classified	Injury_And_Poisoning
998.59	Other_postoperative_infection	Injury_And_Poisoning
